# Thiazoles, Their Benzofused Systems, and Thiazolidinone Derivatives: Versatile and Promising Tools to Combat Antibiotic Resistance

**DOI:** 10.1021/acs.jmedchem.9b01245

**Published:** 2020-03-25

**Authors:** Stella Cascioferro, Barbara Parrino, Daniela Carbone, Domenico Schillaci, Elisa Giovannetti, Girolamo Cirrincione, Patrizia Diana

**Affiliations:** † Dipartimento di Scienze e Tecnologie Biologiche Chimiche e Farmaceutiche (STEBICEF), Università degli Studi di Palermo, Via Archirafi 32, 90123 Palermo, Italy; ‡ Department of Medical Oncology, VU University Medical Center, Cancer Center Amsterdam, DeBoelelaan 1117, 1081HV, Amsterdam, The Netherlands; § Cancer Pharmacology Lab, Fondazione Pisana per la Scienza, via Giovannini 13, 56017 San Giuliano Terme, Pisa, Italy

## Abstract

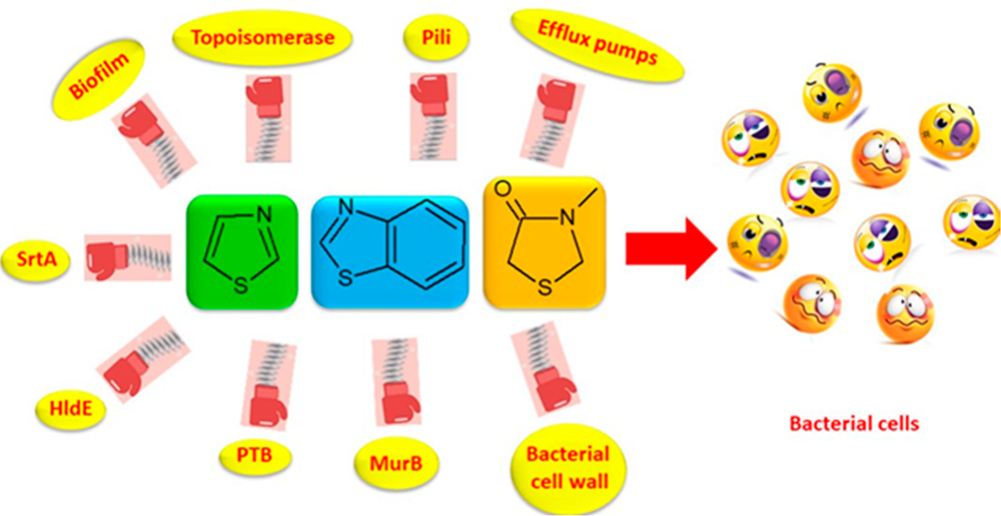

Thiazoles, their benzofused systems, and thiazolidinone derivatives are widely recognized as nuclei of great value for obtaining molecules with various biological activities, including analgesic, anti-inflammatory, anti-HIV, antidiabetic, antitumor, and antimicrobial. In particular, in the past decade, many compounds bearing these heterocycles have been studied for their promising antibacterial properties due to their action on different microbial targets. Here we assess the recent development of this class of compounds to address mechanisms underlying antibiotic resistance at both bacterial-cell and community levels (biofilms). We also explore the SAR and the prospective clinical application of thiazole and its benzofused derivatives, which act as inhibitors of mechanisms underlying antibiotic resistance in the treatment of severe drug-resistant infections. In addition, we examined all bacterial targets involved in their antimicrobial activity reporting, when described, their spontaneous frequencies of resistance.

## 1 Introduction

Antibiotic resistance (AMR) is among the most relevant health problems of this century. At least 700 000 people die of no longer treatable infections every year in the world.^1^

AMR crisis involves any class of antibiotic including the second- and third-line antibiotics once considered the last resort drugs to tackle common infections. We are losing our ability to keep under control many common infections; consequently, one of the pillars on which modern medicine is based threatens to collapse.

The phenomenon of AMR at biochemical and physiological levels may manifest in any single bacterial cell (planktonic growth) or in a sessile complex microbial community (biofilm).^2^ Bacteria might become resistant to antibiotics at the cellular level, and bacteria can become resistant to antibiotics by inactivating or altering the molecular structure of an antibiotic, modifying the antibiotic target, and decreasing the intracellular drug concentration by expressing efflux pumps. These biochemical mechanisms are present in any single bacterial cell embedded in the biofilm structure too, however, a set of adaptive and intrinsic mechanisms due to growth as a community contribute to making biofilms up to a thousand times more resistant than single bacterial cells.^3^

Within a global strategy for fighting antibiotic resistance, in 2005, the World Health Organization (WHO) published for the first time a list called Critical Important Antimicrobials (CIA). This list includes antibiotics and antimicrobials widely used in the world, divided in three categories of clinical importance (critically important, divided in highest priority and high priority; highly important; important) with the scope of preserving the clinical use and the rational employment of these molecules. A recent recommendation of the Interagency Coordination Group on Antimicrobial Resistance (IACG) of the United Nations is to stop the use of antibiotics categorized as the highest priority critically (i.e., third and higher generation cephalosporins, macrolide, quinolones and fluoroquinolones, glycopeptides, macrolides and ketolides, polymyxins) in nonclinical settings (farms, food, and feed production).^4^

There are indeed many scientists who fear that the world is going toward a “post-antibiotic era” where common infections, which were easily cured, could become chronic or fatal. As a consequence, about 10 million deaths caused by infectious diseases treatment failure could be reached in 2050.^5^

In this scenario, the development of new small molecules able to counteract the commonest mechanisms underlying antibiotic resistance including enzymatic inactivation of antibiotics, alterations in cell penetrability, efflux pumps activity or biofilm formation is urgently required.^6,7^

Many heterocyclic compounds have been synthesized in the past decade in the attempt to obtain new antimicrobials, which would be able to treat infections caused by resistant bacterial strains. Herein we focused on synthetic molecules bearing the thiazole, benzothiazole, and thiazolidinone scaffolds, which are widely recognized as nuclei of great value for obtaining molecules endowed with various biological activities,^8^ including analgesic,^9^ anti-inflammatory,^9^ antidiabetic,^10^ antitumor,^11^ and antimicrobial.^12^

Sulfur containing compounds such as thiazole, compared to other five-membered heterocycles such as oxazole and imidazole, possess unique features due to the low lying C–S σ* orbitals that, conferring small regions of low electron density on sulfur (σ-holes), may play a role in drug–target interactions.^13^ The presence of sulfur exerts also significant effect on bond angles and the topology of substituents, it being associated with longer lengths and smaller bond angles. Moreover, thiazole ring systemshows cLogP and cLogD values near to 0.5 and p*K*_a_ and p*K*_BHX_ (the latter related to the H-bond interaction properties) of 2.53 and 1.37, respectively. Although the benzo-fusion of thiazoles causes a significant reduction of p*K*_a_ values, this modification has a limited effect on p*K*_BHX_ values (Table 1).^14^

**Table 1:**
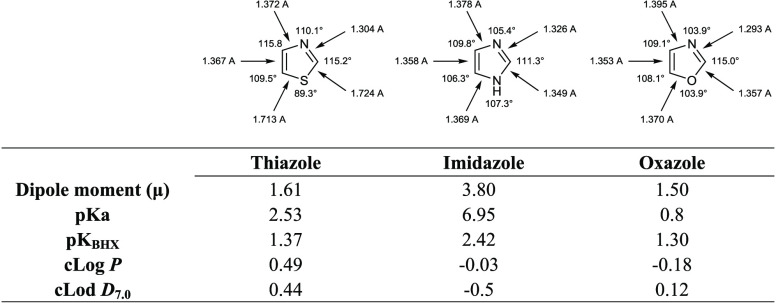
Chemical-Physical Properties of Thiazole, Imidazole, and Oxazole Scaffolds

In particular, we assess the recent development (2008–2019) of this class of compounds in the treatment of antibiotic resistance mechanisms at both bacterial cell and community levels. We also review the SAR and the potential clinical implementation of thiazole and benzothiazole with antibacterial properties in the therapies for severe drug-resistant infections.

## 2 DNA Gyrase and Topoisomerase IV Inhibitors

To obtain more potent therapeutic strategies, many targets were investigated, among them, type II topoisomerase enzymes, which include bacterial DNA gyrase and topoisomerase IV. These enzymes were discovered in 1976 in the Gram-negative pathogen *Escherichia coli* by Gellert and co-workers and are at the center of many studies because of their commercial success with the development of the fluoroquinolones.^15^ Gyrase maintains negative supercoiling of the bacterial chromosome, while topoisomerase IV is responsible for untangling daughter chromosomes. Type II topoisomerases are crucial enzymes for maintaining DNA integrity during the replication playing key roles in DNA replication, transcription, and repair, recombination, and transposition.^16^ One of the most important advantage to consider the bacterial type II topoisomerases as targets for the design of novel therapeutic classes of antibiotics is that they are highly conserved enzymes in bacterial strains and therefore their inhibition could lead to a broad spectrum activity.^17^

Fluoroquinolones represent an important class of dual inhibitors of bacterial type II topoisomerases. Despite their clinical success in the past, their use is now limited by growing antibiotic resistance, which strongly compromised their effectiveness. Both for DNA gyrase and topoisomerase IV, fluoroquinolones, interacting with the enzyme-bound DNA complex, determine conformational modifications preventing the assembly of DNA strands that impede the normal enzyme activity. This causes the inhibition of DNA synthesis and the consequent death of the bacterial cell.

Several searches have been carried out in order to obtain new drugs able to inhibit both enzymes, DNA gyrase and topoisomerase IV, reducing the probability that bacteria develop antibiotic resistance mechanisms.^18^ A special effort must be made in identifying selective inhibitors toward the bacterial form which are inactive against the eukaryotic type II topoisomerases. This desirable selectivity can be achieved because there are distinct structural differences from the homodimeric mammalian enzyme counterparts.

Structurally, DNA gyrase is composed of two heterodimeric subunits GyrA and two GyrB in complex, whereas topoisomerase IV consists of two ParC and two ParE subunits.

**Figure 1:**
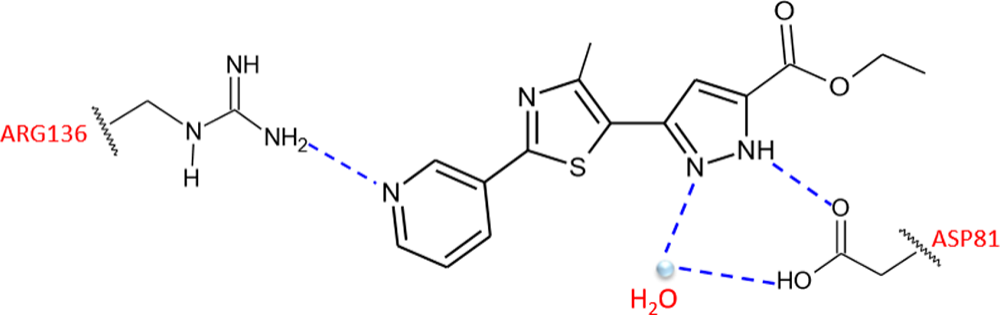
Schematic representation of the binding mode in GyrB active site of *S. aureus* of compound **2e**. Hydrogen bonds are represented as blue dashed line.

With the aim of obtaining specific inhibitors of GyrB, which is considered an interesting target to overcome the cross-resistance to quinolones, Ronkin and collaborators performed a high-throughput screen. This screen identified compound **1** (Table 2), which showed an inhibitory activity against GyrB in *E. coli* with a *K*_i_ value of 2.9 μM.^19,20^ The X-ray crystal structure of this derivative inside the GyrB active site of *Staphylococcus aureus* allowed the identification of structural features responsible for the interactions with the enzyme, guiding the synthesis of the analogues (**1a**–**d** and **2a**–**y**). It was observed that (i) the pyrazole moiety was involved in a hydrogen bond with Asp81 and a highly conserved structural water molecule. (ii) the presence in position 2 of the thiazole ring of a 3-pyridyl moiety (**2e**), assuring an additional hydrogen bond with Arg136, improved the activity by 5–10-fold compared to **1a** and **1d** (Figure 1), (iii) the substituent at the 4 position of the thiazole ring affected the activity of the compound, substituents larger than methyl should be tolerated and lipophilic groups gave better results, (iv) the position 5 of the pyrazole ring can be substituted with carboethoxy, amide, or carbamate groups. In particular, the ethyl amide compounds are twice as potent as the corresponding ethyl esters. Another key feature is the presence of a carbamate group, which was able to form an additional hydrogen bond with Asn46, as observed in derivatives **2t**–**y** (Table 2), which were the most potent in term of enzymatic inhibition showing *K*_i_ values in the range 0.040–3.4 μM. Despite their interesting activity against GyrB, carbamates **2t**–**y** did not show antibacterial activity against wild-type *Escherichia coli* strains as well as *S. aureus* ATCC 29213 and *Streptococcus pneumoniae* ATCC 10015; this is probably due to the efflux pumps antimicrobial resistance mechanism.

**Table 2:**
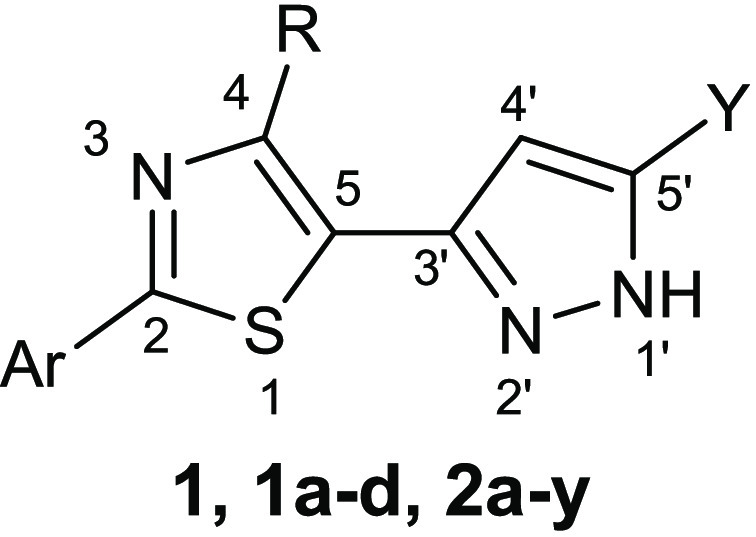

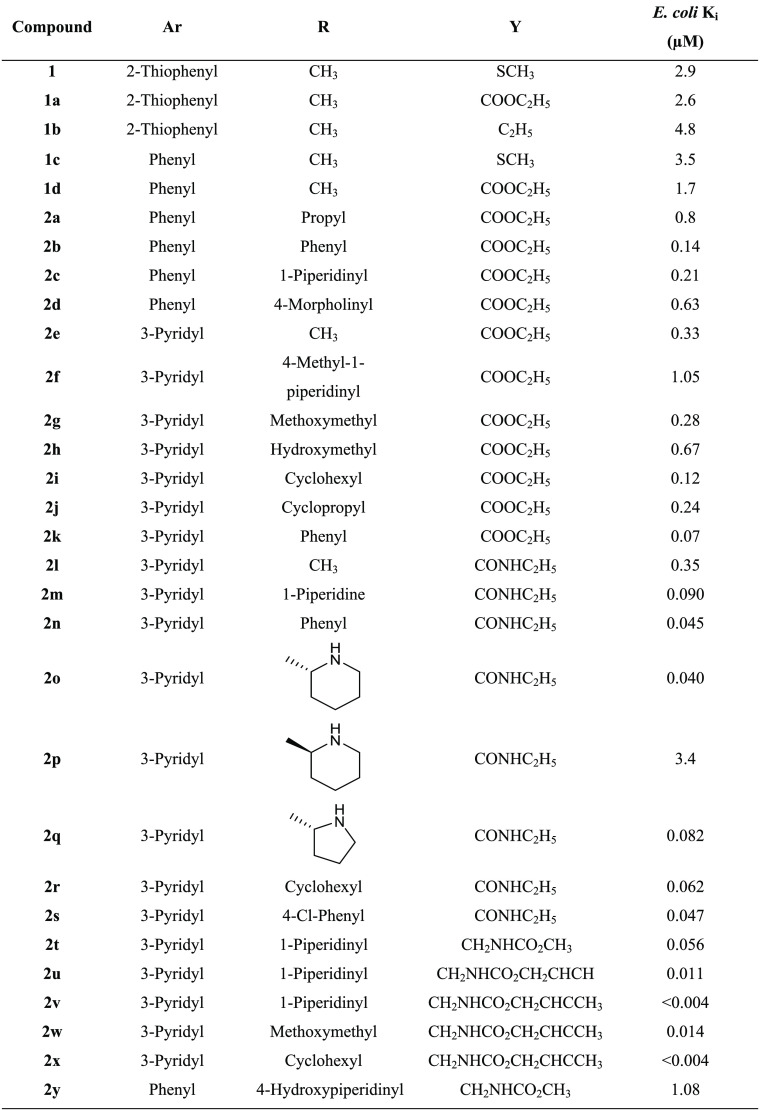
Chemical Structures and *E. coli* GyrB Inhibitory Activity of Compounds **1**, **1a**–**d**, and **2a**–**y**

**Figure 2:**
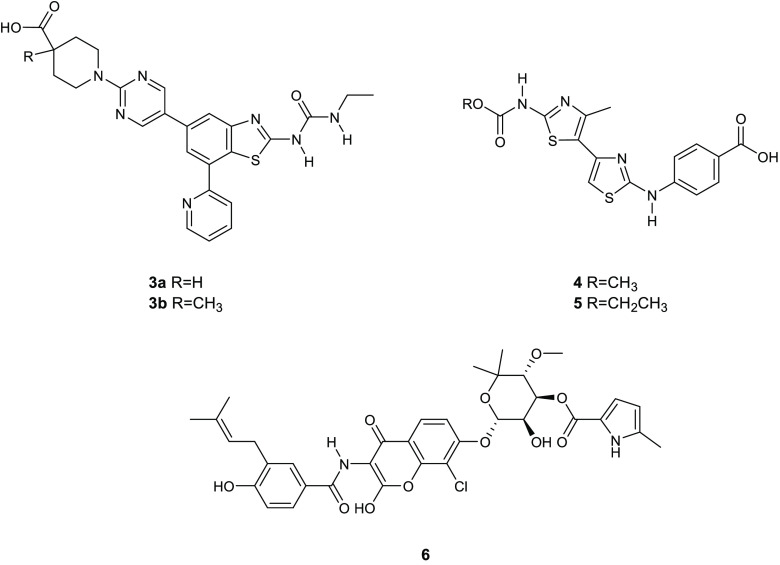
Chemical structures of compounds **3a**,**b**, **4**, **5**, and chlorobiocin (**6**).

In an attempt to discover innovative inhibitors of both GyrB and ParE, Stokes and collaborators synthesized two benzothiazole ethyl urea compounds **3a**,**b** (Figure 2), which potently inhibited in vitro the ATPase activity of *E. coli* DNA gyrase and topoisomerase IV, eliciting IC_50_s in the range from 0.0033 to 0.046 μg/mL.^21^ Derivatives **3a**,**b** were extremely more potent against topoisomerase enzymes than the reference drug novobiocin. Moreover, differently from compounds **1** and **2**, they showed a significant antibacterial activity against a broad range of Gram-positive/negative pathogens with MIC values of 0.008, 0.03, and 0.06 μg/mL against *S. pneumoniae* ATCC 49619, *Staphylococcus epidermidis* ATCC 1228, and *Streptococcus pyogenes* ATCC 51339, respectively.

Compounds **3a**,**b** were also tested for evaluating their spontaneous frequencies of resistance (FoRs). It is known that antibacterial compounds acting on a single bacterial target show FoRs in the range 10^–6^ to 10^–9^,^22^ conversely, the FoR values for the two benzothiazole ethyl urea derivatives **3a**,**b** against *S. aureus* ATCC 29213 were <2.3 × 10^–10^ and <2.3 × 10^–11^, respectively, at concentrations 8-fold the MIC. This behavior seems to be due to the capability of the compounds **3a**,**b** of simultaneously inhibiting two intracellular targets, i.e., GyrB and ParE. Additionally, these compounds proved to be not toxic when assayed against the HepG2 human liver cell line.

It was observed that the presence of a cyclic amine between the carboxylate and the C-5 aryl group enhanced the antibacterial activity and the solubility compared to the derivatives without this group.^23^ Further improvement in the PK profile was obtained with the inclusion of an α substituent to the carboxylic acid, which showed enhanced solubility, excellent oral bioavailability, and lower clearance. Remarkably, compounds **3a**,**b** had powerful inhibitory activity against *S. aureus* topoisomerase IV (0.012 and 0.008 μg/mL, respectively), proving their selectivity toward the bacterial isoform without affecting human topoisomerase II.

Most of the known bacterial topoisomerase inhibitors have been identified with the aid of computational techniques such as virtual screenings and docking studies in the active sites.^24^

Brvar and collaborators employed the crystal structure of the N-terminal portion of the B subunit of DNA gyrase, the G24 protein (PDB 1KZN), cocrystallized with the natural inhibitor, chlorobiocin (**6**) (Figure 2) to generate a structure-based pharmacophore model.^25^ Twelve features involved in the interaction between the inhibitor and the enzyme were identified: (i) the presence of two hydrogen bond donors, (ii) two hydrogen bond acceptors, (iii) an aromatic ring, and (iv) seven hydrophobic portions. This pharmacophore was successfully validated by assessing its ability to recognize the bioactive conformation of **6**, and then was used to perform a large scale virtual screening of 5 million compounds, which allowed the identification of 400 new hit compounds belonging to different classes.

Further in silico studies, including a docking calculation for the potential interactions of the G24 active-site with the binding site for ATP, resulted in the identification of new strong antigyrase agents with 4,5′-bithiazole-2,2′-diamine structure. Among these agents, compound **4** (Figure 2) had the most promising DNA gyrase inhibitory activity displaying an IC_50_ of 5.5 μM.

**Figure 3:**
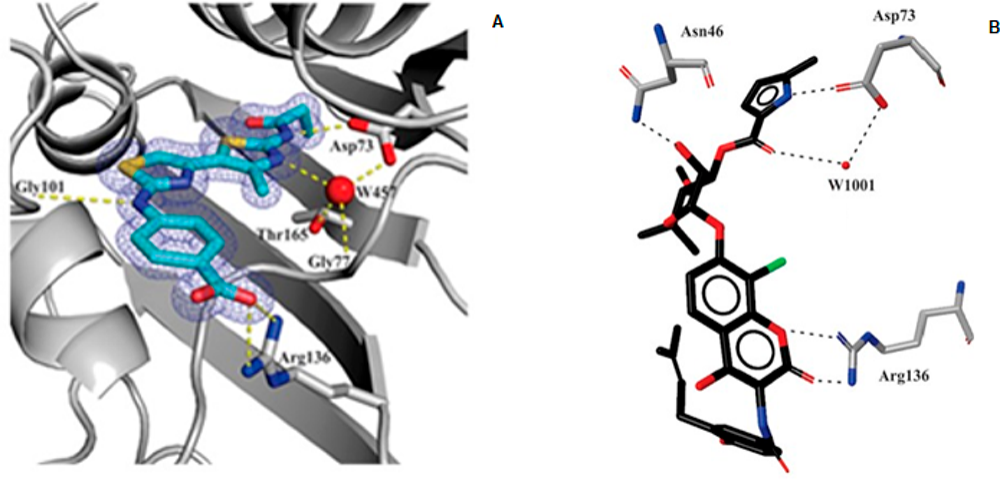
(A) Bithiazole **5** in G24 active site. Hydrogen bonds are designed as dotted lines; electronic density, contoured at 2σ, is reported as meshed net. (B) Chlorobiocin (**6**) binding mode into the G24 ATP-binding.^25^Reproduced with permission from ref (25). Copyright 2012 American Chemical Society.

Subsequently, a commercially available library of 240 4′-methyl-*N*2-phenyl-[4,5′-bithiazole]-2,2′-diamines was docked into the G24 ATP-binding site, using the software GOLD. This study focused on compounds capable to bind the previously selected residues Asp73, Thr165 through W1001 water molecule and Arg136. Eight compounds were selected to be examined for their inhibitory activity on DNA gyrase inhibitory activity, and the bithiazole **5** (Figure 2) emerged for its potency, showing an IC_50_ of 1.1 μM. The binding mode of this derivative was confirmed by X-ray crystallography (Figure 3), and its structure has been reported in the RCSB Protein Data Bank (PDB 4DUH).^25^ Despite their structural diversity, compound **5** and the coumarin inhibitor chlorobiocin (**6**) showed a very similar binding pattern. As observed for **6**, the bithiazole **5** formed two hydrogen bonds with (1) Asp73, through the 2′-propionylamido group, which was oriented in the active site as the 5-methylpyrrole ring of **6**, and (2) the conserved water molecules that were coordinated with the residues Asp73, Gly77, and Thr165. The propionyl moiety participated also to the hydrophobic interactions with the binding pocket made by Val43, Val71, Val120, and Val 167. This is the same binding pocket, occupied by the methylpyrrole group of **6**. Additionally, the second thiazole ring was oriented in a manner analogous to the methyl groups of the sugar part of **6** and formed hydrophobic interactions with the residues Ile78, Ile90, and Val120. The 2-aminothiazole moiety seems essential in the DNA gyrase inhibition as it contains an acceptor–donor interaction pattern, which is a fundamental requirement for this activity. Similarly, the presence of a hydrogen bond acceptor on the para position of the phenyl ring seems to play a key role in the enzymatic activity.

An important class of gyrase inhibitors is constituted by marine alkaloids, which are widely recognized as molecules of great value in terms of biological properties and many derivatives in the past decade have been successfully developed for different therapeutic purposes.^26−28^

However, the marine alkaloids oroidin analogues have attracted great attention for their significant antibacterial and antibiofilm activities.^29−32^

**Figure 4:**
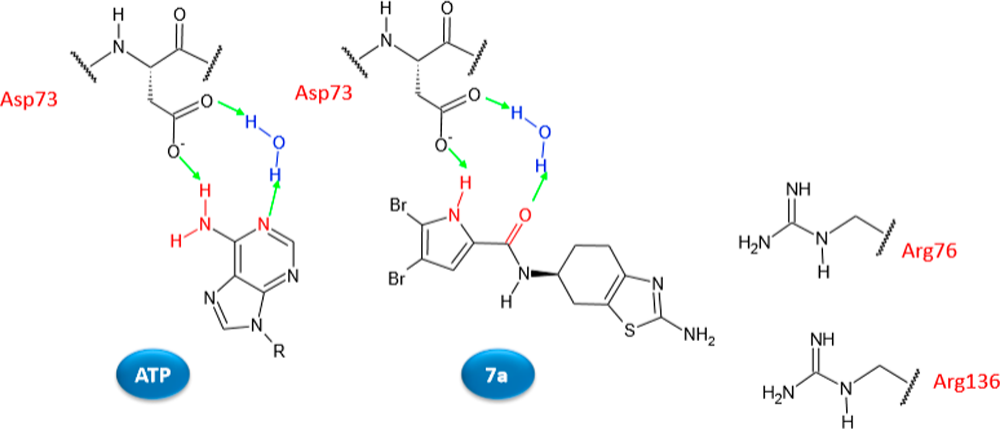
Schematic representation of the binding mode in the *E. coli* GyrB ATP-binding site of ATP and compound **7a**. Hydrogen bonds are represented as green arrows.

Performing a virtual screening of a library of oroidin analogues employing the *E. coli* GyrB crystal structure (PDB 4DUH), Tomǎsič and co-workers identified a 4,5,6,7-tetrahydrobenzo[1,2-*d*]thiazole derivative **7a** (Table 3) able to inhibit *E. coli* DNA gyrase with an IC_50_ of 12 μM.^33^ To better understand how to optimize the structure of the compound **7a** in order to obtain more potent inhibitors, its binding mode in the ATP-binding site of *E. coli* GyrB (PDB 4DUH) was investigated. The 4,5-dibromo-1*H*-pyrrole-2-carboxamide moiety is involved in the interaction with the hydrophobic pocket and is able to make hydrogen bonds with the Asp73 side chain as well as with the conserved water molecule, miming ATP interactions with the same residue, whereas the position of the 2-amino group on the thiazole ring allows substitution with functional groups, such as oxalyl, malonyl, and succinyl (Figure 4), for interaction with Arg76 and/or Arg136.

**Table 3:**
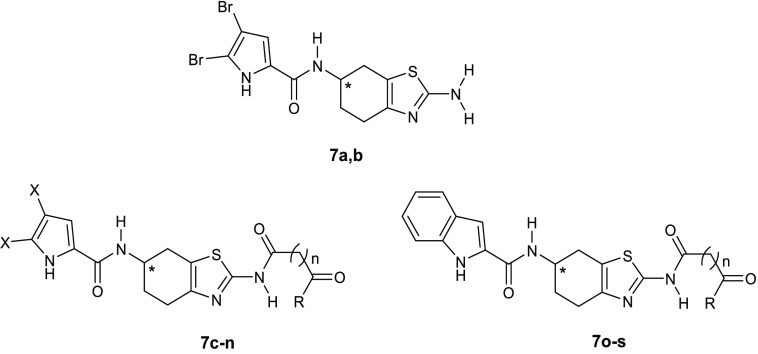
Inhibitory Activity of *E. coli* and *S. aureus* DNA Gyrase and Topoisomerase IV by the 4,5,6,7-Tetrahydrobenzo[1,2-*d*]thiazoles Containing the 4,5-Dibromo-1*H*-pyrrole (**7a**–**h**), 4,5-Dichloro-1*H*-pyrrole Moiety (**7i**–**n**), or 1*H*-Indole Moiety (**7o**–**s**)

					DNA gyrase IC_50_ (*K*_d_^a^) [μM] or RA [%]^b^		topoisomerase IV IC_50_ (*K*_d_^a^) [μM] or RA [%]^b^		*E. coli* MIC [μg/mL]		
compd	*	R	X	n	*E. coli*	*S. aureus*	*E. coli*	*S. aureus*	*wt*	*tolC*	*impA*
neg^c^					100%	100%	100%	100%			
NB^d^					0.17 μM	0.040 μM	11 μM	27 μM			
**7a**	*S*				12 μM	90%	101%	102%	>256	64	64
**7b**	*R*				47 μM	118%	96%	92%	>256	64	64
**7c**	*S*	OC_2_H_5_	Br	0	0.10 μM	80 μM	74%	180 μM	>256	128	>256
**7d**	*S*	OCH_3_	Br	1	0.096 μM	110 μM	86 μM	74%	>256	16	32
**7e**	*S*	OCH_3_	Br	2	0.093 μM	113%	97%	99%	>256	32	64
**7f**	*S*	OH	Br	0	0.058 μM	120 μM	200 μM	78 μM	>256	256	>256
**7g**	*S*	OH	Br	1	0.069 μM	86 μM	74 μM	76 μM	>256	256	>256
**7h**	*S*	OH	Br	2	0.049 μM	270 μM	90%	110 μM	>256	256	>256
**7i**		OC_2_H_5_	Cl	0	0.40 μM	320 μM	300 μM	290 μM	>256	256	>256
**7j**		OCH_3_	Cl	1	0.40 μM	63%	97%	101%	>256	32	32
**7k**		OCH_3_	Cl	2	0.89 μM	86%	100%	93%	>256	>256	>256
**7l**		OH	Cl	0	0.59 μM	300 μM	82%	170 μM	>256	>256	>256
**7m**		OH	Cl	1	0.13 μM	87%	98%	102%	>256	>256	>256
**7n**		OH	Cl	2	0.30 μM	10 μM	72%	97%	>256	>256	>256
**7o**		OC_2_H_5_		0	10 μM	76%	101%	99%	nt	nt	nt
**7p**		OCH_3_		2	9.1 μM	87%	101%	104%	nt	nt	nt
**7q**		OH		0	7.7 μM	79%	47%	99%	>256	>256	128
**7r**		OH		1	7.6 μM	117%	96%	103%	>256	>256	>256
**7s**		OH		2	8.1 μM	95%	99%	79%	>256	>256	>256

a *K* determined by surface plasmon resonance.

b Residual activity of the enzyme at 100 μM concentration of the tested compound. nt: not tested.

Many analogues were prepared to get more insights into the SAR of the 4,5,6,7-tetrahydrobenzo[1,2-*d*]thiazole class, as well as to improve the affinity toward the enzyme. These studies also evaluated the effect of the replacement of the 4,5-dibromo-1*H*-pyrrole with the 4,5-dichloro-1*H*-pyrrole or 1*H*-indole moieties. In addition, experiments with the (*R*)-isomer (**7b**) of compound **7a** assessed the influence of chirality on enzymatic inhibition.

All the synthesized compounds were tested against *E. coli* DNA gyrase. Then the compounds with IC_50_s lower than 50 μM were evaluated for their inhibitory activities against *S. aureus* DNA gyrase as well as *E. coli* and *S. aureus* topoisomerases IV (Table 3).

The substituents on the pyrrole ring play a pivotal role in the activity of these compounds. This might be explained by the hydrophobic interactions of the bromine atoms with the pocket of the ATP-binding site. The introduction of an oxalyl, malonyl, or succinyl group, as a hydrogen bond acceptor, led to derivatives (**7c**,**d**,**e**), as suggested by computational studies. These compounds enhanced the inhibition of *E. coli* DNA gyrase, with IC_50_s of 0.10, 0.096, and 0.093 μM, respectively, probably due to their capacity to interact with Arg136. The stereochemistry seems also to have an important role in determining activity (*S*-isomers, in fact, showed stronger inhibitory activity than the (*R*)-counterparts.

In the indole series (compounds **7o**–**s**), the activity was maintained in the low micromolar range. Conversely, the substitution of the bromine with chlorine atoms on the pyrrole ring (compounds **7i**–**n**) led to a decrease inpotency toward DNA gyrase as well as topoisomerase IV.

Unfortunately, despite the activity showed by compounds **7** in the enzymatic assay, no derivative of the series showed antibacterial activity at the concentration of 50 μM against the Gram-negative *E. coli* ATCC 25922, *P. aeruginosa* ATCC 27853, and the Gram-positive *E. faecalis* ATCC 29212 and *S. aureus* ATCC 25923 bacterial strains. Only compounds **7d** against *E. faecalis* and **7e** and **7j** against *S. aureus* displayed percentages of growth inhibition higher than 50%.

To investigate if the lack of antibacterial effects against the Gram-negative pathogens of this class was ascribable to permeability problems or to efflux pumps, the 4,5,6,7-tetrahydrobenzo[1,2-*d*]thiazoles **7** were tested against permeabilized (*impA*) and efflux pump knockout (*ΔtolC*) strains of *E. coli*. However, among the tested compounds, only five derivatives, **7a**, **7b**, **7d**, **7e**, and **7j**, showed better antibacterial effects toward *impA* and *ΔtolCE. coli* strains than the wild-type.

Even if DNA gyrase is a fundamental enzyme for the bacterial viability, often it was observed that its inhibition in a biochemical assay does not correspond to an antibacterial effect at the cellular level.

Structural modifications of the most active *E. coli* DNA gyrase inhibitor **7f**, which showed an IC_50_ value of 58 nM, led to two series of nanomolar inhibitors **8a**–**e** (Table 4) and **9a**–**g** (Table 5).^34,35^ In particular, the replacement of the 4,5,6,7-tetrahydrobenzo[1,2-*d*]thiazole scaffold with a planar benzothiazole-2,6-diamine moiety and the move of the 4,5-dibromopyrrole-2-carbonyl group from N-6 to N-2 of the central core proved to be advantageous for the enzymatic activity. The most potent inhibitor was the derivative **8d**, which displayed an IC_50_ of 38 nM (Table 4). However, even if the DNA gyrase inhibitory activity increased, no improvement in the antibacterial properties was observed, probably because efflux pumps inactivated these compounds. The compounds, in fact, proved to be significantly more potent toward the efflux deficient strain, *ΔtolCE. coli* than the wild type.

**Table 4:**
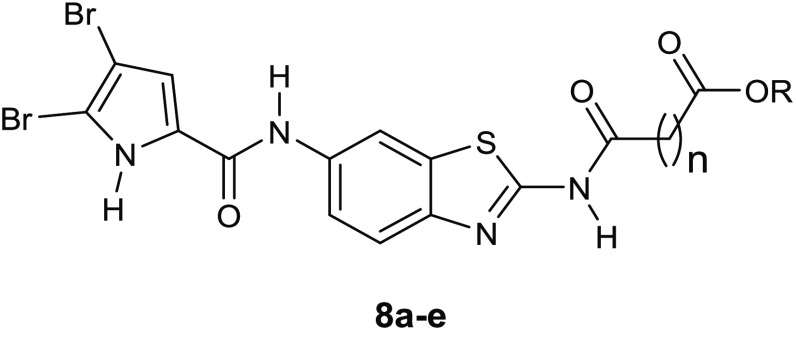
Inhibition of *E. coli* and *S. aureus* DNA Gyrase and Topoisomerase IV by the Benzothiazole Compounds with the Pyrrole-2-carboxamido Moiety at Position 6 on the Benzothiazole Core (**8a**–**e**)

			DNA gyrase IC_50_ [μM]		topoisomerase IV IC_50_ [μM]	
compd	n	R	*E. coli*	*S. aureus*	*E. coli*	*S. aureus*
novobiocin			0.17	0.040	11	27
**8a**	0	CH_2_CH_3_	0.081 ± 0.05	>100	>100	>100
**8b**	1	CH_3_	0.24 ± 0.02	>100	>100	>100
**8c**	2	CH_3_	0.87 ± 0.21	>100	>100	>100
**8d**	0	H	0.038 ± 0.001	>100	5.4 ± 1.0	2.5 ± 0.2
**8e**	2	H	0.057 ± 0.018	>100	4.5 ± 0.7	2.2 ± 0.3

**Table 5:**
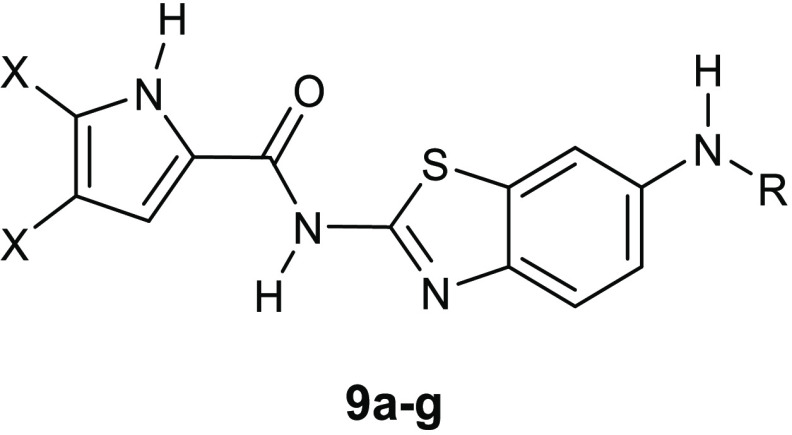

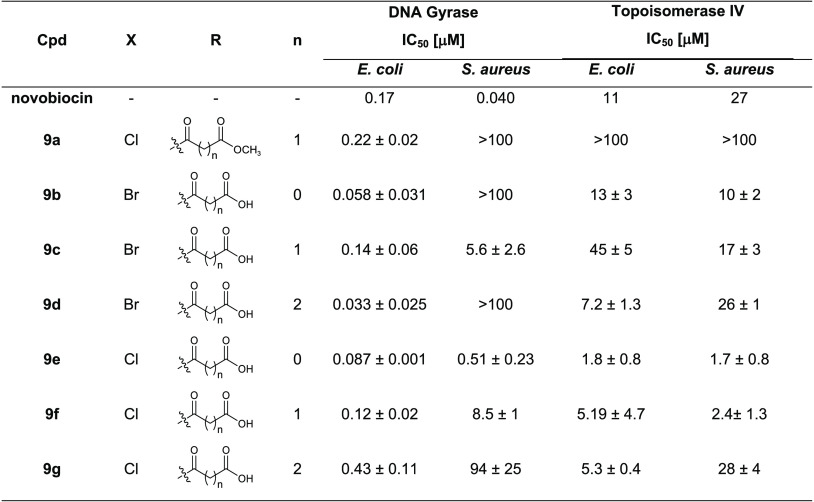
Inhibition of *E. coli* and *S. aureus* DNA Gyrase and Topoisomerase IV by the Benzothiazole Compounds with the Pyrrole-2-carboxamido Moiety at Position 2 on the Benzothiazole Core (**9a**–**g**)

For the compounds of the series **9**, the highest activity was reached against the *E. coli* DNA gyrase by the derivative **9d**, which showed an IC_50_ value against this enzyme of 33 nM (Table 5).

**Figure 5:**
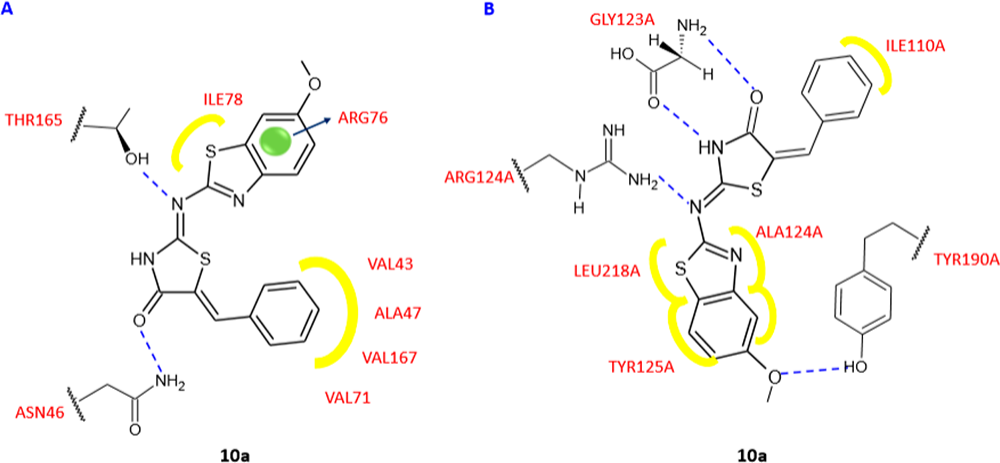
Binding mode of compound **10a** in the topoisomerase II DNA gyrase active site (1KZN) (A) and binding mode of compound **10a** in the *E. coli* Mur B active site (2Q85) (B). Yellow, van der Waals interactions; blue dashed line, hydrogen bond; blue arrow, π interaction.

Taking into account the interesting antibacterial properties described for benzothiazole and thiazolidinone scaffolds, Haroun and collaborators synthesized a new series of compounds **10a**–**l** (Table 6), bearing both bioactive nuclei. All compounds were tested for their activity against the Gram-negative *E. coli* (ATCC 35210), *Enterobacter cloacae*, *Pseudomonas aeruginosa* (ATCC 27853), *Salmonella typhimurium* (ATCC 13311) pathogens, as well as against the Gram-positive *Listeria monocytogenes* (NCTC 7973), *Bacillus cereus* (clinical isolate), *Micrococcus flavus* (ATCC 10240), and *S. aureus* (ATCC 6538). All of these novel derivatives showed good antibacterial potency, proving to be more active than reference drugs, such as ampicillin and streptomycin, with MIC values in the range of 0.0018–0.28 μmol/mL and MBC values in the range of 0.0036–0.37 μmol/mL. An initial hypothesis on the mode of action of these compounds came from an evaluation of docking studies conducted on topoisomerase II DNA gyrase (1KZN) and *E. coli* Mur B (2Q85) (Figure 5A,B), showing that these compounds were capable of binding to the active sites of both enzymes, with a specific affinity toward MurB. In particular, the best binding score and binding energy values toward both enzymes were found for compound **10a**, which interacted with the binding site of topoisomerase II DNA gyrase through two hydrogen bonds, between the nitrogen atom of the central chain and the Thr165 and between the keto group of thiazolidinone ring and Asn46. Moreover, the phenyl ring is involved in hydrophobic interactions with Val71, Val167, Ala47, and Val43. The greater affinity toward MurB observed for derivative **10a** was due to the ability of this compound to form four hydrogen bonds with the active site, two with Gly123, and the other two with Arg124 and Tyr190. In addition, the benzothiazole scaffold formed hydrophobic interactions with Val129, Leu218, and Ala124. MurB is a UDP-*N*-acetylenolpyruvoylglucosamine reductase, which leads the catalysis the second committed reaction in the biosynthesis of peptidoglycan, and therefore it is fundamental for bacterial growth. However, assays on the enzyme were not conducted to confirm the computationally obtained data, therefore the target of this series remains unknown. Additionally, it should be useful to investigate the potential of compounds **10a**–**l** as pan assay interference compounds (PAINS), because many subclasses of aminothiazoles were classified in this category and, therefore, could give false positive results.^36^

**Table 6:**
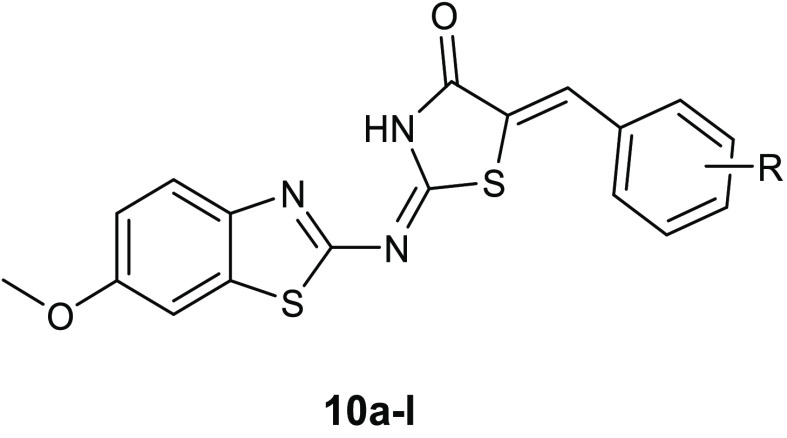
Chemical Structures and Antibacterial Activity of Compounds **10a**–**l**

		MIC values (MBC values) μmol/mL × 10^2^							
compd	R	*B. cereus*	*M. flavus*	*S. aureus*	*L. monocytogenes*	*E. coli*	*E. cloacae*	*P. aeruginosa*	*S. typhimurium*
**10a**	H	1.49 (2.97)	2.97 (5.94)	1.19 (1.49)	1.49 (2.97)	0.23 (2.97)	1.49 (2.97)	4.45 (5.94)	1.49 (2.97)
**10b**	4-OH	4.90 (9.80)	9.80 (19.6)	4.90 (9.80)	4.90 (9.8)	9.80 (19.6)	4.90 (9.80)	4.90 (9.80)	4.90 (9.80)
**10c**	4-OCH_3_	4.80 (19.2)	9.60 (19.2)	4.80 (9.60)	9.60 (19.2)	14.4 (19.2)	7.20 (9.60)	9.60 (19.2)	4.80 (9.60)
**10d**	4-OH; 3-OCH_3_	4.60 (9.20)	13.9 (18.6)	9.20 (37.3)	18.6 (37.3)	13.9 (18.6)	6.90 (9.20)	9.20 (37.3)	3.45 (4.60)
**10e**	2-Cl	4.80 (9.60)	9.60 (19.2)	4.80 (9.60)	9.60 (19.2)	9.60 (19.2)	4.80 (9.60)	9.60 (19.2)	4.80 (9.60)
**10f**	3-Cl	4.60 (9.20)	4.60 (9.20)	4.60 (9.20)	4.60 (9.20)	0.18 (0.36)	4.60 (9.20)	4.60 (9.20)	4.60 (9.20)
**10g**	4-Cl	4.80 (19.2)	9.60 (19.2)	4.80 (9.60)	9.60 (19.2)	9.60 (19.2)	4.80 (9.60)	9.60 (19.2)	2.40 (4.80)
**10h**	2-NO_2_	9.10 (18.2)	9.10 (18.2)	9.10 (18.2)	4.50 (9.10)	9.10 (18.2)	4.50 (9.10)	4.50 (9.10)	9.10 (18.2)
**10i**	3-NO_2_	14.0 (18.4)	18.4 (36.8)	14.0 (18.4)	18.4 (36.8)	23.3 (36.8)	23.3 (36.8)	18.4 (36.8)	4.60 (9.20)
**10j**	4-NO_2_	0.58 (1.16)	9.20 (18.4)	0.58 (1.16)	9.20 (18.4)	9.20 (18.4)	2.30 (9.20)	9.20 (18.4)	0.58 (1.16)
**10k**	4-OH; 3,5-OCH_3_	8.80 (17.6)	8.80 (17.6)	4.40 (8.80)	2.20 (8.80)	8.80 (17.6)	4.40 (8.80)	2.20 (8.80)	2.20 (8.80)
**10l**	4-OH; 3-OCH_3_; 5-I	7.20 (28.8)	7.20 (14.4)	3.60 (7.20)	28.8 (36.0)	7.20 (14.4)	3.60 (14.4)	14.4 (28.8)	7.20 (14.4)

**Figure 6:**
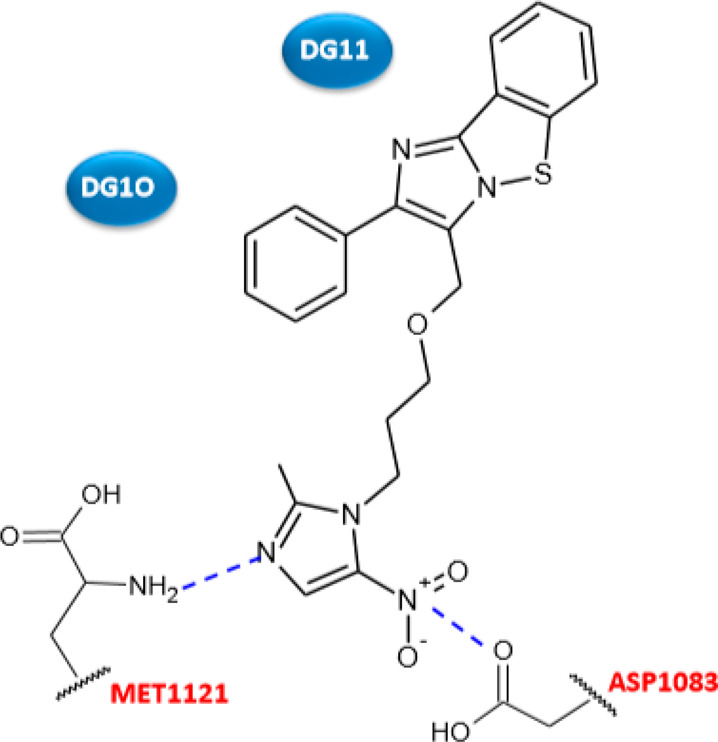
Schematic representation of the binding of compound **11d** into the active site of *S. aureus* DNA gyrase. Hydrogen bonds in blue dashed lines.

Azoalkyl ether imidazo[2,1-*b*]benzothiazoles **11a**–**j** (Table 7) were described as antibacterial drugs with a wide spectrum of activity against both Gram-positive and Gram-negative pathogens, showing in some cases higher potency than the reference drugs chloromycin (**12**) and norfloxacin (**13**).^37^ In particular, compound **11d** with a propyl linker was up to 8-fold more effective against methicillin-resistant *S. aureus* (MRSA) and *S. typhi* eliciting a MIC values of 2 and 1 μg/mL, respectively. To evaluate the capability of derivative **11d** to stimulate bacterial resistance, a time-kill kinetic experiment was performed on MRSA. The results highlighted an instant bactericidal effect against MRSA without effects on the onset of antibiotic resistance. The possible mechanism of action was investigated through (i) a docking study aiming to evaluate the interaction of derivative **11d** with the binding site of *S. aureus* DNA gyrase (PDB 2XCS) and (ii) a UV–vis spectroscopic analysis to clarify the binding mode with MRSA DNA. The computational study disclosed the possibility of compound **11d** to interact with the complex of gyrase–DNA by π–π stacking between the two carbonyl groups of the imidazo[2,1-*b*] benzothiazole backbone and base pairs DG-11 and DG-10 of DNA. The nitrogen atom of the azole cycle was able to form a hydrogen bond with Met1121, whereas the nitro group bond to residue Asp1083 (Figure 6). Additionally, UV–vis spectroscopy showed that compound **11d** is capable to enter into MRSA–DNA, preventing the replication process of DNA.

**Table 7:**
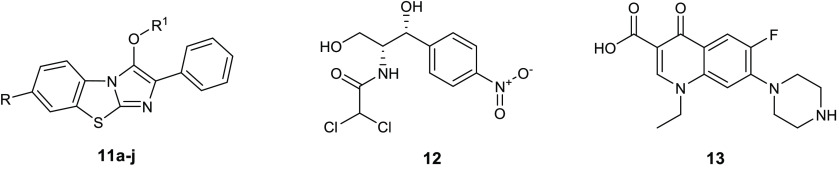

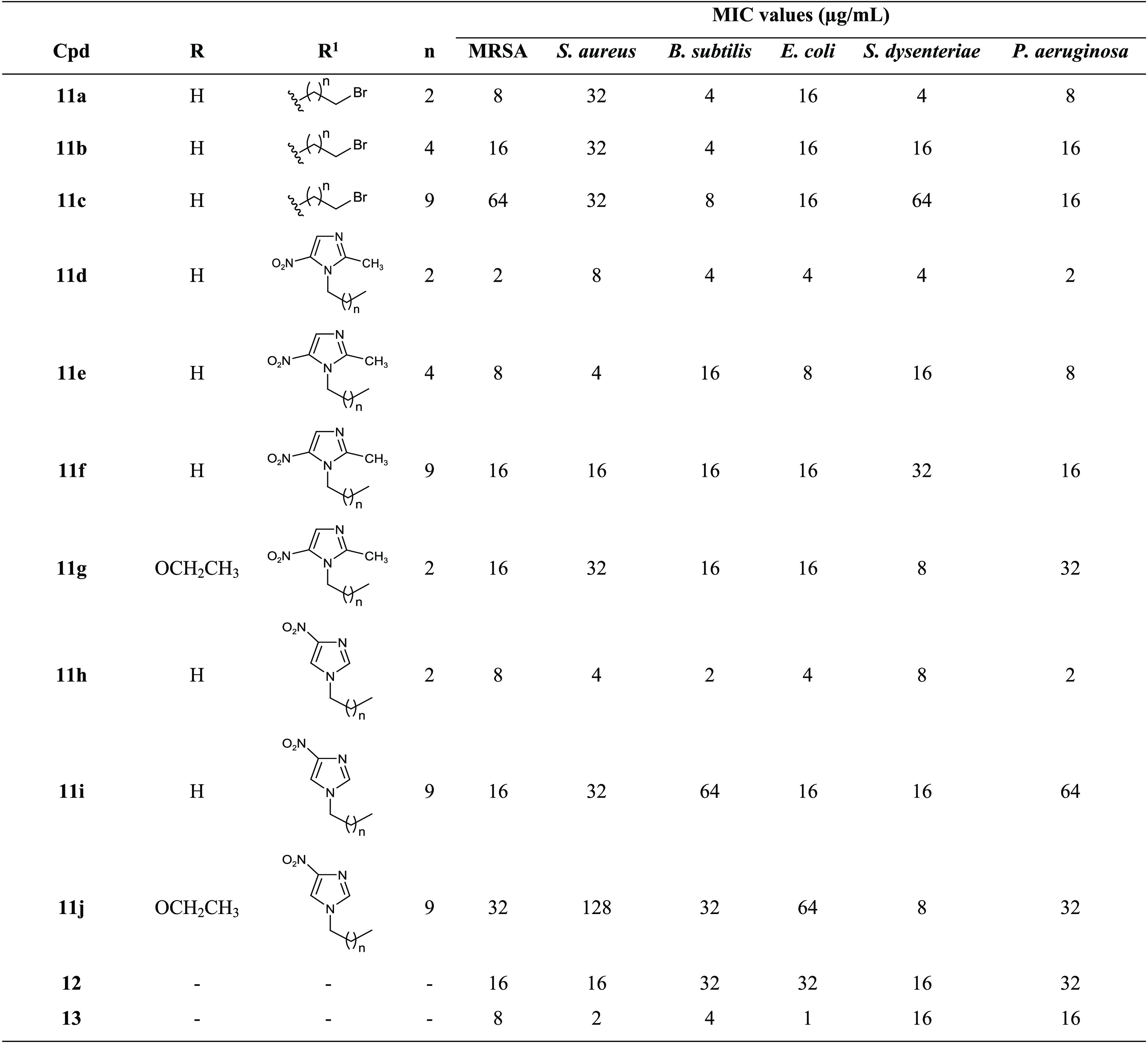
Chemical Structures and Antibacterial Activity of Compounds **11a**–**j**, Chloromycin (**12**), and Norfloxacin (**13**)

Schiff bases constitute a class of compounds endowed with a broad spectrum of biological activity, probably ascribable to the presence of the electrophilic carbon atom and the nucleophilic nitrogen atom of the imine group, which may provide precious binding sites for the interaction with the biological target. Schiff bases also demonstrate to be sufficiently stable in in vitro and in vivo assays.^38^ On the basis of this knowledge, a series of Schiff bases of thiazolyl-triazole **14a**–**g** (Table 8) were reported as antibacterial compounds and potential DNA gyrase inhibitors.^39^ All of these compounds were evaluated against the Gram-positive *S. aureus* ATCC 49444 and *Listeria monocytogenes* ATCC 19115 and the Gram-negative *P. aeruginosa* ATCC 27853 and *S. typhimurium* ATCC 14028. *L. monocytogenes* was the most susceptible strain, with MIC values of 1.9–3.9 μg/mL, which are lower than ciprofloxacin (**15**). Among the Gram-negative, *P. aeruginosa* was potently inhibited by derivatives **14a**–**g** (MIC = 1.9 μg/mL). The ratio between MBC and MIC disclosed a bactericidal effect for these molecules. Docking studies on the DNA gyrase from *L. monocytogenes* suggested a mechanism of action in which this enzyme is involved. In particular, all the Schiff bases seem to prevent the entry of *O*-(5′-phospho-DNA)-tyrosine intermediate in the binding site.

**Table 8:**
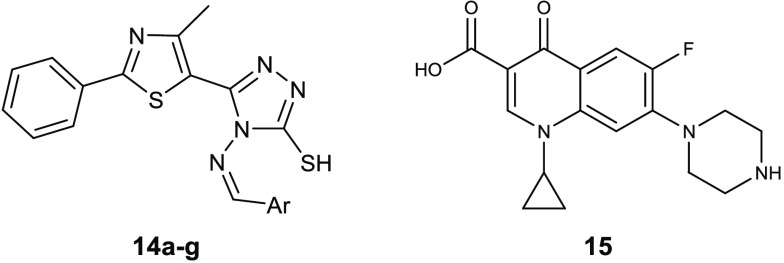
Chemical Structures and Antibacterial Activities of Compounds **14a**–**g** and Ciprofloxacin (**15**)

		MIC (μg/mL)			
compd	Ar	*S. aureus* ATCC 49444	*L. monocytogenes* ATCC 19115	*P. aeruginosa* ATCC 27853	*S. typhimurium* ATCC 14028
**14a**	4-Br-phenyl	31.2	1.9	1.9	62.5
**14b**	4-F-phenyl	31.2	1.9	1.9	62.5
**14c**	4-NO_2_-phenyl	62.5	1.9	1.9	62.5
**14d**	2-OCH_3_-phenyl	31.2	1.9	1.9	62.5
**14e**	3-OCH_3_-phenyl	31.2	1.9	1.9	31.2
**14f**	2-thienyl	15.6	1.9	1.9	62.5
**14g**	4-(CH_3_)_2_*N*-phenyl	31.2	1.9	1.9	62.5
**15**		1.9	3.9	3.9	0.97

**Figure 7:**
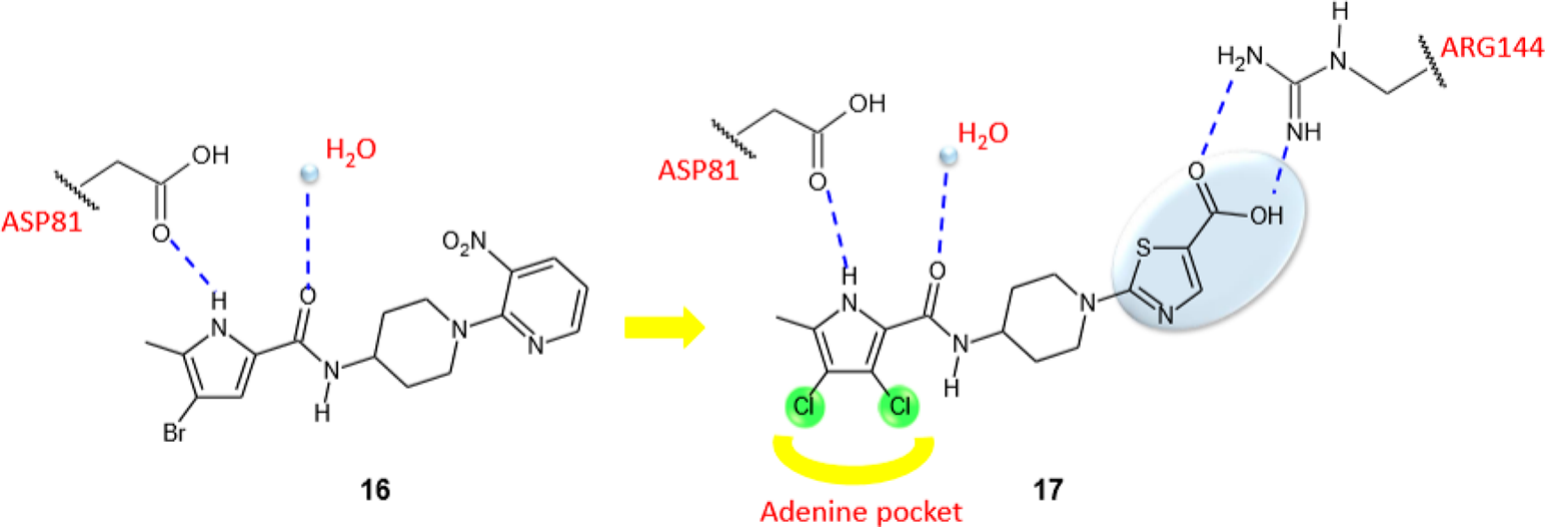
Graphic representation of the binding mode of compounds **16** and **17**. Hydrogen bonds are represented with dashed blue lines, while hydrophobic interactions are in yellow.

Eakin et al. identified a series of pyrrolamides with potent DNA gyrase inhibitory activity, employing a fragment-based lead generation approach (FBLG) with NMR screening to discover compounds with low molecular weight able to bind the ATP-binding pocket of *E. coli* GyrB.^40^ Starting from almost 1000 compounds with molecular masses ranging from 100 to 370 Da, the pyrrole **16** (Figure 7) showed a DNA gyrase IC_50_ of 3 μM and was chosen as the lead compound for the design of more potent inhibitors. The structural optimization of pyrrolamide **16** had mainly the purpose of improving the antibacterial activity of this class of compounds. In fact, despite its potent inhibitory activity against the enzyme, the compound **16** was ineffective in vitro against the tested bacterial strains (*E. coli* ARC523, *E. coli* ARC524, *Haemophilus influenzae* KW20, *S. aureus* ARC516, *S. pneumoniae* ARC548, *Enterococcus faecium* ARC521). Structural modifications of pyrrolamide **16**, suggested by computational and X-ray crystallography studies, created a powerful GyrB inhibitor, incorporating the thiazole nucleus, **17** (Figure 7), endowed with a significant antibacterial activity against *S. pneumoniae* using a mouse model of lung infection. The crystallographic structure of derivative **16** inside the ATP-binding domain of *S. aureus* GyrB revealed a common interaction pattern with the natural substrate, in particular, the pyrrole moiety occupied the same pocket of the ATP adenine, creating a hydrogen bond with Asp81, whereas, the carbonyl group was involved in a hydrogen bond with a conserved water molecule.

The introduction in compound **17** of the two chlorine atoms in the pyrrole moiety proved to be particularly advantageous for the affinity toward the enzyme. These atoms indeed increased the hydrophobic interactions with the adenine pocket and because of their electron withdrawing effect and improved the ability of the pyrrole NH group in establishing a hydrogen bond with the Asp81. Moreover, the presence of a carboxyl group on the thiazole ring enhanced the affinity toward the enzyme, forming two additional hydrogen bonds with the Arg144 of the active site. Pyrrolamide **17** showed MBC values against *S. aureus*, *H. influenzae*, and *S. pneumoniae* of 8, 4, and 0.5 μg/mL, respectively, eliciting no cytotoxicity toward mammalian cells.

The ability of **17** to induce spontaneous resistance was evaluated at the concentration of 8 μg/mL in *S. aureus* ARC516 and a frequency of 2.5 × 10^–9^ was observed. Importantly, in vivo efficacy of compound **17** assessed in an *S. pneumoniae* lung infection model in mice (1 × 10^5^ CFU/lung) has been found at the oral dose of 160 mg/kg.

**Figure 8:**
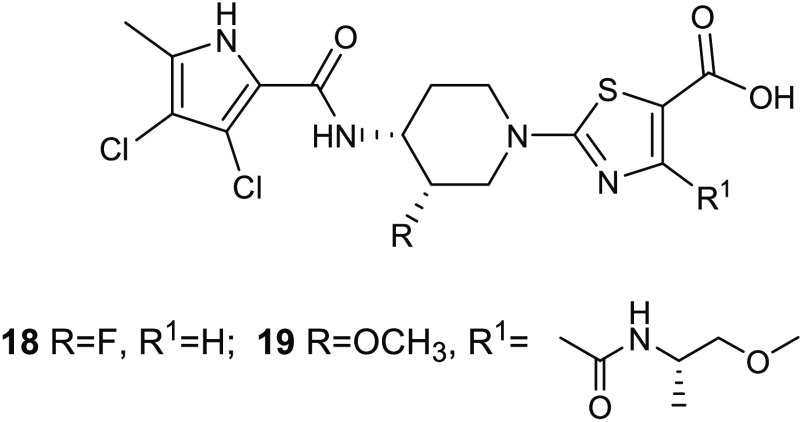
Chemical structures of compound **18** and AZD5099(**19**).

**Figure 9:**
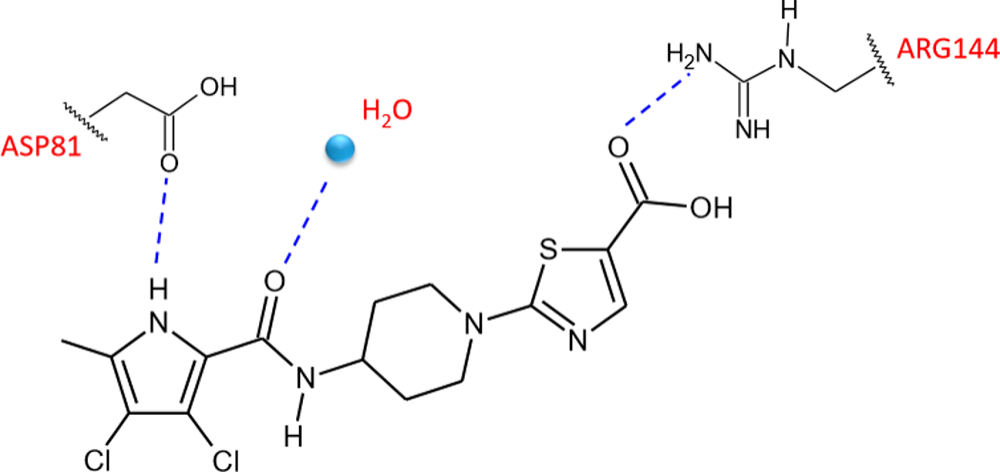
Representative model of the binding mode of compound **18** in complex with *S. aureus* GyrB (3TTZ) illustrated by ball and stick design.

The fluorine atom on the 3-piperidine position characterizes **18** (Figure 8), which was obtained as mixture of four possible distereoisomers, including the (3*S*,4*R*)-distereoisomer, which showed enhanced inhibitory activity against DNA gyrase and antibacterial potency. The presence of fluorine atom, which occupied a region close to the hydrophobic pocket of the enzyme, determined an improvement in the activity by 4–8-fold. This might be due to a modification in the piperidine conformation, which allowed stronger interactions of the pyrrole carboxamide and the thiazole carboxylate with the Asp81 and Arg84, respectively. Additionally, the carboxyl group of the thiazole ring is involved in a salt bridge with Arg144 (Figure 9). This new enantiomerically pure thiazole analogue showed improved in vivo potency against *S. pneumoniae* in a mouse model of pneumonia.^41^ The compound was orally administered 18 h postinfection with 10^5^ CFU/lung of *S. pneumonia* ARC548, and the in vivo activity was evaluated through viable counts in dilutions of lung homogenates. A dose-dependent reduction in viable bacterial counts in the lung was observed, eliciting the highest response at the dose of 80 mg/kg.

Further SAR studies, carried out with purpose of optimizing the structure of these compounds, resulted in the identification of the antibacterial agent AZD5099 (**19**) (Figure 8), which entered phase I clinical trials as a novel treatment of Gram-positive and Gram-negative infections.^42^ The replacement of the fluorine atom at the 3-position of the piperidine scaffold with a methoxyl group was particularly advantageous in terms of enzyme inhibitory activity and antibacterial properties. Additionally, the carboxylic group in the thiazole nucleus was crucial for the activity because it is involved in a salt bridge interaction with the residue Arg144. AZD5099 (**19**) proved to be efficacious in different in vivo models, in particular, for cure of nosocomial lung and skin infective diseases caused by relevant Gram-positive such as *S. aureus*. Studies on the pharmacokinetic and bioavailability highlighted the suitability of AZD5099 (**19**) for both parenteral and oral administration.

## 3 Modulators of Bacterial Cell Wall Synthesis and Permeability

### 3.1 MurB Inhibitors

The MurB enzyme is an NADPH dependent UDP-*N*-acetylenolpyruvylglucosamine reductase that plays a key role in the second step of bacterial peptidoglycan biosynthesis.

**Figure 10:**
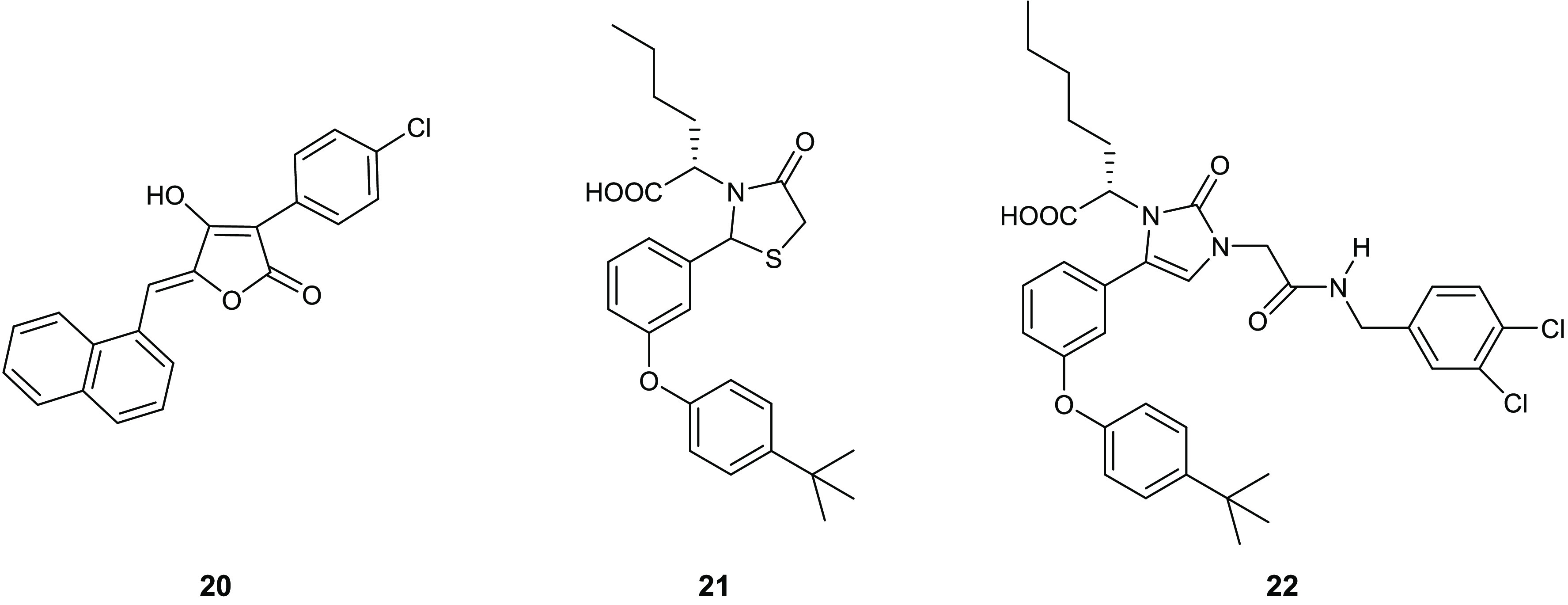
Representative examples of small molecules with MurB inhibitory activity.

In particular, the Mur pathway enzymes MurA and MurB catalyze the synthesis of UDP-*N*-acetylmuramic acid (UDP-MurNAc) from UDP-*N*-acetylglucosamine (UDP-GlcNAc). Subsequently, MurC, MurD, MurE, and MurF add amino acids to UDP-MurNAc and generate the UDP-MurNAc-pentapeptide, which is incorporated into the nascent bacterial peptidoglycan.^43^ MurB emerged as an interesting target for novel antibacterial drugs for many reasons: (i) its inhibition leads to a bactericidal effect because it is fundamental for the bacterial growth, (ii) there is no analogue in the eukaryotic cell therefore the selectivity should be easily obtainable, and (iii) being present in both Gram-positive and Gram-negative pathogens its inhibition should result in a broad spectrum antibacterial activity (Figure 10).^44^

Among the thiazole compounds, 4-thiazolidinones, which are the tetrahydro derivative of thiazole bearing a carbonyl group, are widely described in recent years as they present different biological activities.^45−50^

The ability of the 4-thiazolidinone scaffold in inhibiting this enzyme was first described in 2000 by Andres and co-workers, who identified three derivatives **20**–**22** endowed with potent *E. coli* MurB inhibitory activity at low micromolar level (Figure 10).^51^ Among them, the 4-thiazolidinonederivative **21** results the most potent compoundwith an IC_50_ value of 7.7 μM, which is 2-fold lower than those obtained by derivatives **20** and **22** (IC_50_ values of 14 and 15 μM, respectively). It was hypothesized, as supported by in silico studies reported by the authors, that 4-thiazolidinone scaffold may act as MurB inhibitor by mimicking the diphosphate group.

### 3.2 Penicillin Binding Protein Inhibitors

A series of thiazolidinone derivatives **23a**–**e** (Table 9) was synthesized and examined for inhibitory activity toward transpeptidases and β-lactamase.^52^

**Table 9:**
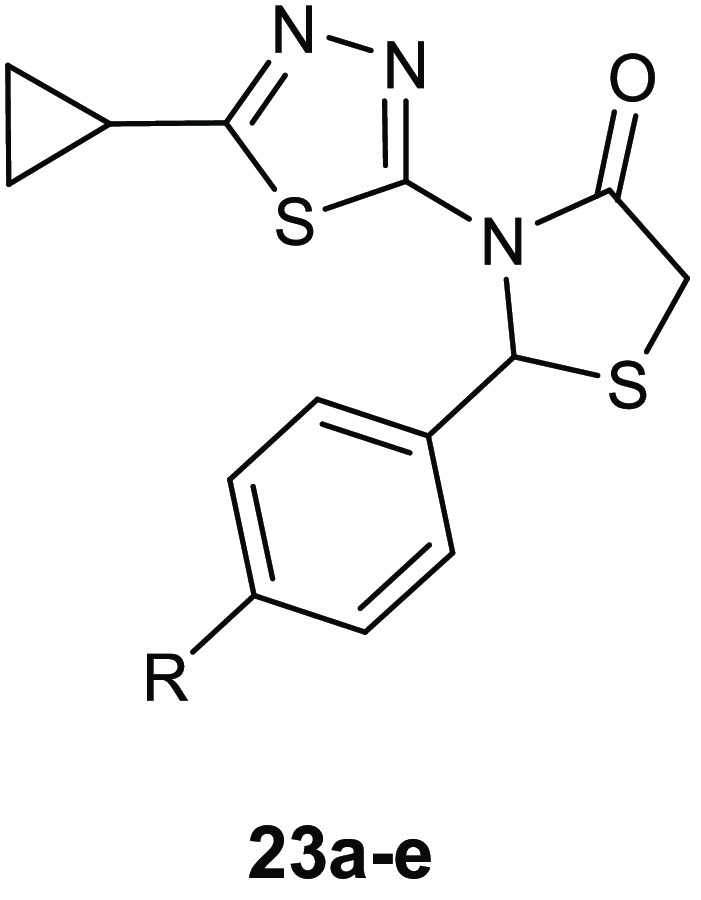
Chemical Structures and Antibacterial Activity of Compounds **23a**–**e**

		MIC values (μg/mL)			
compd	R	*S. aureus* ATCC 25923	*B. subtilis* ATCC 6633	*E. coli* ATCC 25922	*P. aeruginosa* ATCC 27853
**23a**	Cl	31.25	31.25	125	125
**23b**	CN	62.50	62.50	250	250
**23c**	OCH_3_	125	125	250	250
**23d**	N(CH_3_)_2_	250	250	500	500
**23e**	NO_2_	15.60	31.25	62.50	125

When assayed against the Gram-positive bacteria *S. aureus* ATCC 25923 and *Bacillus subtilis* ATCC 6633, the Gram-negative bacteria *E. coli* ATCC 25922, and *P. aeruginosa* ATCC 27853 compounds **23a**–**e** showed MIC values within the range of 15.60–500 μg/mL, exhibiting a strong selectivity toward Gram-positive pathogens. The most active derivative **23e** elicited MIC values of 15.60, 31.25, and 62.50 μg/mL against *S. aureus*, *B. subtilis*, and *E. coli*, respectively. Studies on the mechanism of action were carried out by employing the method of Heipieper for evaluating the leakage of UV_260_ and UV_280_ (mostly nucleic acid material and protein) absorbing material. In these studies, **23e** was able to alter the membrane permeability causing cell death. In addition, docking studies into the active site of penicillin binding protein (PBP) (PDB 1FXV) and β-lactamase (PDB 4FH2) enzymes highlighted a higher affinity for the PTB transpeptidase, supporting the hypothesis of a mechanism of inhibition of the synthesis of peptidoglycan by inhibiting the enzyme PTB transpeptidase. The compound showed a binding pattern similar to that of ampicillin, forming a hydrogen bond with the key residue of the binding site Ser 1B. The determination of the leakage of UV_260_- and UV_280_-absorbing material, which mainly consists of nucleic acid material and protein, confirmed that the compound acts by altering membrane permeability.

The authors utilized for their docking studies the conformation reported in Table 9 instead of the preferred topology involving the C=O oxygen atom proximal to S stabilized by an O to S interaction.^13,14^ Therefore, the authors have jeopardized the reliability of the obtained results.

Further biochemical studies on the enzyme should be carried out to confirm this hypothetical mechanism of action and for validating the docking results.

**Figure 11:**
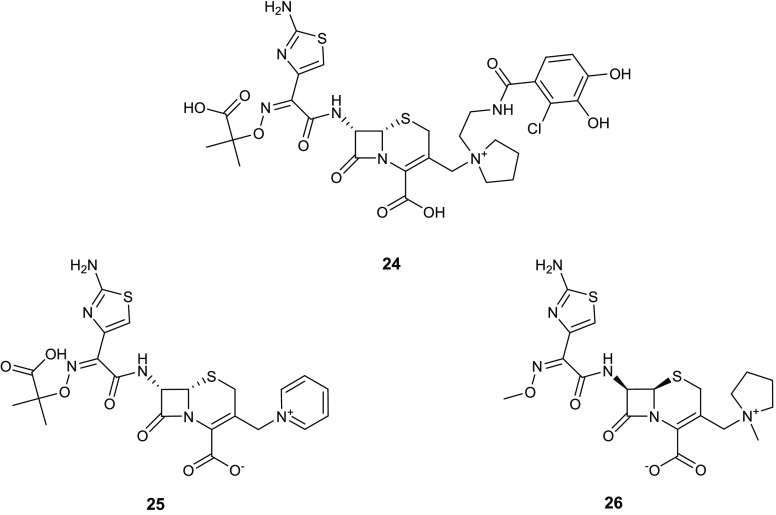
Chemical structures of cefiderocol (**24**), ceftazidime (**25**), and cefepime (**26**).

The thiazole scaffold is also present in a novel injectable siderophore cephalosporin bearing a catechol moiety, known as cefiderocol (**24**) (Figure 11), which demonstrated powerful antibacterial activity against Gram-negative pathogens. This activity is caused by the inhibition of penicillin binding proteins and thus interfering with the bacterial cell wall synthesis.^53,54^ Cefiderocol (**24**), although structurally very similar to the third- and fourth-generation cephalosporins ceftazidime (**25**) and cefepime (**26**) (Figure 11), elicited higher stability toward different β-lactamases such as AmpC and extended-spectrum β-lactamases.

The key role of the aminothiazole ring, common to many broad-spectrum cephalosporins, in enhancing the activity against Gram-negative bacteria is well described. Thus, high resolution crystal structures of the *P. aeruginosa* PBP3 with many compounds including marketed β-lactams, as well as in silico studies, highlighted that ceftazidime (**25**) affinity for B3-PBPs was due to the interaction of its aminothiazole ring with the binding pocket.^55^ The amino acid residues of the aminothiazole binding pocket are conserved in many B3-PBPs. Therefore, the presence of aminothiazole, determining an increase in the affinity toward these proteins, usually is responsible of the enhancement of activity against Gram-negative bacteria.^56^ In fact, none of the many cephalosporin derivatives synthesized using different heterocyclic systems instead of aminothiazole ring showed comparable potency.^57^ Additionally, the carboxypropyl-oxyimino group is responsible for the increased stability of the compound toward β-lactamases hydrolysis. Of note, the presence of a catechol group on the side chain improved significantly the periplasmic concentrations of cefiderocol (**24**), with respect to the other cephalosporins, because of its ability to chelate iron, which makes possible the use of iron transportation systems present in the outer membrane (OM) of Gram-negative pathogens (“Trojan horse strategy”).

Cefiderocol (**24**) proved to be efficacious against serious Gram-negative infections, showing a strong activity against infections caused by carbapenem-resistant and MDR Gram-negative bacteria, including MDR Enterobacteriales, *P. aeruginosa*, *Acinetobacter baumannii*, and *Stenotrophomonas maltophilia*.^58^ This compound is being investigated in phase III clinical studies. Previous clinical studies highlighted the efficacy and safety of intravenous cefiderocol (**24**) in patients with complicated urinary tract infections (cUTIs) and in the treatment of many Gram-negative infections, such as healthcare-associated pneumonia, hospital-acquired pneumonia, and ventilator-associated pneumonia.^59^

### 3.3 Inhibitors of Heptose Synthesis

Lipopolysaccharide (LPS) is a highly acylated saccharolipid, which constitutes the main component of the outer leaflet of the OM of Gram-negative pathogens. LPS has a crucial role in maintaining the barrier function by avoiding the passive diffusion of hydrophobic solutes such as antibiotics and detergents into the cell.^60^

LPS is formed by lipid A, a core oligosaccharide and a repeating polysaccharide O-antigen. The core oligosaccharide is constituted by an inner core of 3-deoxy-d-manno-oct-2-ulosonic acid (Kdo) and heptose residues and an outer core of hexoses derivatives.

Inhibition of heptose synthesis leads to vulnerable bacteria with incomplete LPS, which are more sensitive to host defenses and to many antibiotics because of their increased membrane or cell wall permeability. The identification of small molecules interfering with this process is currently recognized as a valuable goal for the development of novel antivirulence agents against Gram-negative pathogens because heptose is not fundamental for Gram-negative survival, but the inhibition of its synthesis causes an attenuation of virulence.

The bacterial synthesis of heptose in *E. coli* involves many enzymes including the transketolase TktA, the ketose–aldose isomerase GmhA, the kinase HldE, the phosphatase GmhB, and the epimerase HldD.^61^ Desroy and collaborators focused their attention on the first step of heptose synthesis, looking for molecules that can inhibit the kinase activity of *E. coli* HldE.^62^

**Scheme 1:**
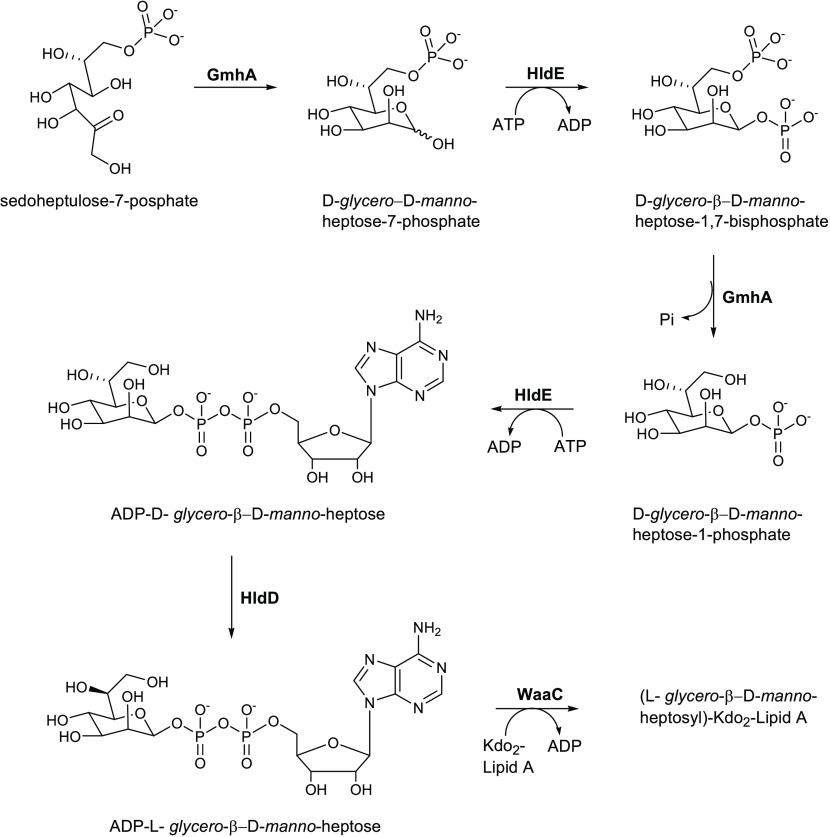
Synthesis of ADP-l-*glycero*-β-d-*manno*-heptose from Sedoheptulose-7-phosphate and Its Incorporation into LPS

HldE in *E. coli* is a bifunctional cytoplasmic ATP-dependent kinase, which uses its two functional domains (carbohydrate kinase and adenylyltransferase) to catalyze the transformation of d-*glycero*-d-*manno-*heptose-7-phosphate in d-*glycero*-β-d-*manno-*heptose-1,7-bisphosphate and of d-*glycero*-β-d-*manno*-heptose-1-phosphate in ADP-D-*glycero*-β-d-*manno*-heptose (Scheme 1).^63^

HldE can be considered a promising target for obtaining antimicrobial agents against Gram-negative pathogens. Because this enzyme is well conserved among bacteria and there are not homologues in humans, the synthesis of selective inhibitors with low toxicity should be feasible. However, heptose synthesis inhibitors are ineffective toward pathogens which have a LPS without heptose such as *Acinetobacter*, *Chlamydia*, and *Moraxella*.^64^

**Figure 12:**
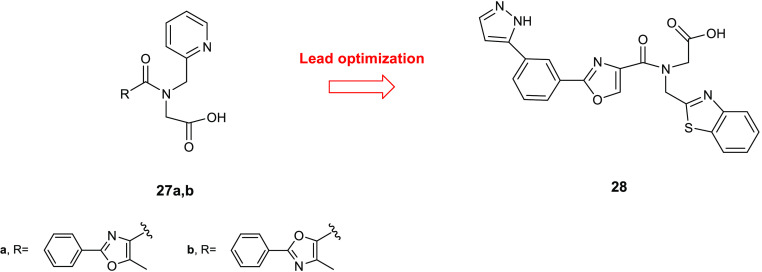
Chemical structures of compounds **27a**,**b** and **28**.

Through a high-throughput screen of 40 000 compounds performed on HldE-kinase activity of *E. coli*, two new inhibitors **27a**,**b** (Figure 12) were identified. Compounds **27a**,**b** showed IC_50_ values of 51 and 69 μM, respectively, behaving as competitive reversible inhibitors toward ATP in the reaction catalyzed by HldE kinase. SAR studies of these compounds led to the identification of more potent derivatives bearing the benzothiazole scaffold. The effect on the HldE kinase activity of various substituents on the phenyl ring was evaluated, and it was observed that (i) the presence of an amino group in *ortho* or *para* position was particularly advantageous for the activity, while, in contrast, it caused a loss of potency in *meta* position, (ii) a nitro group in *ortho* or in *para* position was detrimental for the activity, whereas it proved to be favorable in *meta*, (iii) trifluoromethyl and methoxy groups afforded an improvement in the activity in *meta* position but caused significant reduction in activity in *ortho* and *para* and, finally, (iv) a bromine atom was advantageous for the activity in any position. The replacement of the phenyl ring with heteroaromatic rings or with an ethyl group led to inactive compounds, highlighting the key role of the phenyl moiety for the activity on HldE kinase. In contrast, the oxazole core could be substituted with other heterocycles including thiazole, furan, or pyrazole without any effect on the activity. These results underlined also the importance of the carboxylic acid on the amide side chain because its replacement with other groups, such as amide, alcohol, acylsulfonamide, or tetrazole, led to inactive derivatives. On the basis of these findings and of the observation that the *meta* substitution of the phenyl ring was also relevant for the activity, a derivative **28** (Figure 12), bearing a pyrazole ring in that position, was synthesized and evaluated for its activity against HldE kinase. Among the derivatives of this series, compound **28** elicited the highest activity with an IC_50_ against the enzyme of 0.11 μM.

The inhibitory activities against HldE-kinase and ribokinase (RK) of *E. coli* and HldA of *Neisseria meningitidis* (the equivalent of HldE-kinase in *E. coli*), which share a very similar ATP binding site, were also compared and the results showed a significant selectivity toward HldE/HldA enzymes of Gram-negative bacteria. These compounds are able to interfere with heptose synthesis, altering the barrier function of the LPS but showing no effects on *E. coli* viability, displayed the typical profile of an antivirulence agent. Such compounds, acting on virulence factors without interfering with the bacterial life cycle, impose low selective pressure for the development of bacterial antibiotic resistance mechanisms.

**Figure 13:**
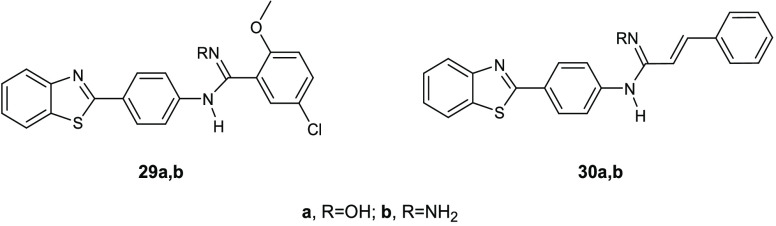
Chemical structures of compounds **29a**,**b** and **30a**,**b**.

Other benzothiazole derivatives acting as membrane perturbing agents able to depolarize the cytoplasmatic membrane of *S. aureus* and *E. coli* are compounds **29a**,**b** and **30a**,**b** (Figure 13).^65^ When assayed for their antibacterial activity against *S. aureus* (ATCC 25323), *E. coli* (ATCC 35218), *P. aeruginosa* (ATCC 27893), *K. pneumonia* (ATCC 31488), *E. faecalis* (clinical isolate), and *S. typhi* (MTCC 3216), the benzothiazole **29a**, bearing a chlorine atom and a methoxy group on the phenyl ring, demonstrated the highest potency with MIC values in the range of 3.91–31.2 μg/mL. The effects of this series in perturbing the permeability of *S. aureus* and *E. coli* membrane was evaluated by using the cationic membrane potential-sensitive cyanine dye, which allows a determination of the alteration of membrane potential and the formation of pores, caused by the compounds, by an increase in fluorescence intensity. Compounds **29a**,**b** were able to damage the membrane structure in *S. aureus* and *E. coli* causing the dispersion of the bacterial cell contents. It was observed that, after interaction with the membrane, compounds **29a**,**b** and **30a**,**b** entered into the cytoplasm and bound to bacterial DNA enhancing the antibacterial effect due to the membrane permeabilization.

## 4 Tryptophanyl-tRNA Synthetase Inhibitors

The aminoacyl-tRNA synthetases are enzymes with important roles in RNA translation because they catalyze the aminoacylation reaction by covalently linking an amino acid to its corresponding tRNA.^66^ These enzymes are essential in bacterial growth and survival because they are involved in many metabolic and signaling pathways essential for cell viability.^67^

Compounds that are able to selectively inhibit the bacterial aminoacyl-tRNA synthetases without interfering with their mammalian analogues can be useful candidates for the development of new classes of antimicrobial agents.

**Figure 14:**
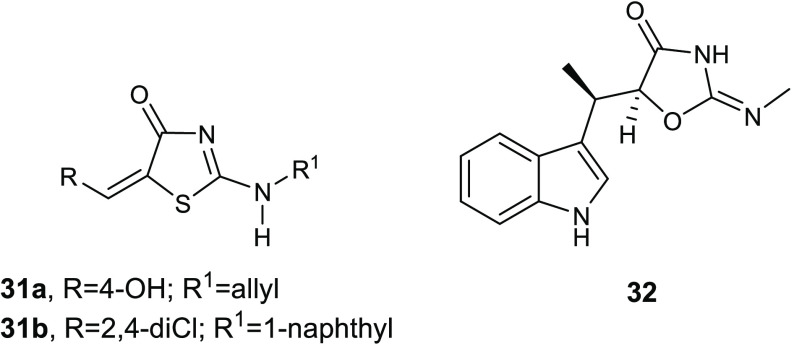
Chemical structures of compounds **31a**,**b** and of the known TrpRS inhibitor indolmycin (**32**).

Recently, Stana and co-workers reported a series of thiazolin-4-one derivatives **31** (Figure 14) endowed with moderate to good antibacterial activity against the Gram-negative bacterial strain *E. coli* ATCC 25922 and the Gram-positive bacterial strain *S. aureus* ATCC 49444.^68^ All compounds were more effective against Gram-positive pathogens, and compounds **31a** and **31b** showed MIC and MBC values against *S. aureus* ATCC 49444 of 0.97 and 1.95 μg/mL, respectively. Because of the structural similarity between compounds **31a** and **31b** with the known tryptophanyl-tRNA synthetase (TrpRS) inhibitor indolmycin (**32**), the authors hypothesized a mode of action involving TrpRS inhibition.

The affinity of the new compounds toward the TrpRS from *S. aureus* (1I6K_P67592) and *E. coli* (5 V0I) was evaluated in silico through molecular docking studies. Results revealed a good affinity of the thiazolin-4-ones due to the formation of polar contacts between exocyclic secondary amine group and the carbonyl group from the thiazolin-4-one ring with amino acid residues of the binding site of the enzyme. Most of the synthesized compounds showed, in silico, better affinity toward TrpRS than the reference compound indolmycin (**32**). The best binding affinity was observed for the compounds incorporating voluminous moieties, such as the α-naphthylamino group, at position 2 of the thiazolin-4-one ring.

Molecular docking studies suggested the key role of the thiazolin-4-one scaffold in polar interactions with the TrpRS binding site. Most compounds showed a common binding pattern involving the carbonyl group of the thiazolin-4-one in the case of *S. aureus* TrpRS. Additionally, the nitrogen atom of the thiazolinone ring hypothesized to be responsible of a polar interaction with the Gly9 residue of the binding site of the most active derivative **31b** against *E. coli*.

## 5 Sortase A (SrtA) Inhibitors

SrtA is a cysteine transpeptidase which anchors the surface proteins, MSRAMMs, to the peptidoglycan of the bacterial cell wall in Gram-positive pathogens. After the recognition of the LPXTG motif in MSRAMMs, SrtA catalyzes the following reactions: (i) thioesterification in which the enzyme cleaves the LPXTG between Thr and Gly, leading to the synthesis of thioester acyl-enzyme intermediate and (ii) the transpeptidation, which allows the formation of a bond between the C-terminal Thr residue of the protein to pentaglycine cross-bridges. The pivotal role of this enzyme in bacterial adhesion and bacterial pathogenesis is well documented and represents therefore an excellent potential target for antivirulence drug development because it is involved in the adhesion of bacteria to host tissues and in biofilm formation, but it is not essential for bacterial viability.^69^ Additionally, SrtA is a membrane enzyme, therefore much more easily accessible than intracellular targets, and there are no analogues of this enzyme in humans so SrtA inhibitors should have low toxicity and high selectivity.

A new series of 2-phenylthiazole derivatives **33a**–**h** (Table 10) were synthesized as SrtA inhibitors.^70^ The antimicrobial activity of the new compounds was tested against five Gram-positive and two Gram-negative bacterial strains. All of the compounds showed good activity against *S. aureus*, in which the lowest MIC value (16 μg/mL) was observed for derivative **33f**. The highest potency was reached by the chlorophenyl derivative **33h** toward *Staphylococcus saprophyticus* ATCC 15305 (MIC = 2 μg/mL).

**Table 10:**
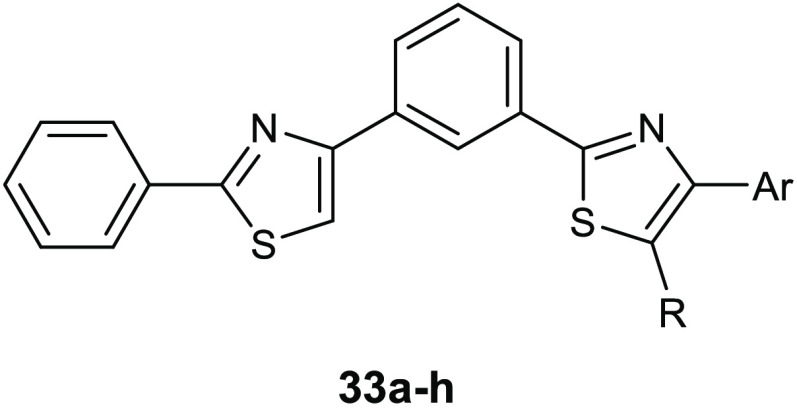
Chemical Structures, Antibacterial Activities, and Antibiofilm Activities of Compounds **33a**–**h**

			MIC (mg/mL)		BIC (mg/mL)
compd	Ar	R	*E. faecalis*	*S. saprophyticus*	*E. faecalis*
**33a**	Ph	CH_3_	0.25	>1.0	0.004
**33b**	Ph	H	0.125	0.032	0.002
**33c**	4-NO_2_-Ph	H	0.062	0.032	0.002
**33d**	4-OCH_3_-Ph	H	0.062	0.125	0.004
**33e**	4-CN-Ph	H	0.062	0.016	0.002
**33f**	1-naphthalene	H	0.016		0.004
**33g**	3-CONH_2_,4-OH-Ph	H	0.25		0.008
**33h**	4-Cl-Ph	H	0.032	0.002	0.016

Because the inhibition of SrtA is correlated to the inhibition of biofilm formation in Gram-positive pathogens, the derivatives were also evaluated for their antibiofilm properties against all the tested strains.

These compounds proved to be more potent against *E. faecalis*, showing BIC_50_, which is defined as the lowest concentration of compound that showed 50% inhibition on the *biofilm* formation, in the range 2–16 μg/mL. Aiming at corroborating the mechanism of action involving SrtA, molecular docking studies evaluated the active sites of SrtA from both bacterial species. Results highlighted higher affinity toward *E. faecalis* SrtA and a common binding pattern, which involved the thiazole nucleus in polar contacts through the nitrogen atoms with the hydroxyl group of Thr122. The presence of polar substituents on the phenyl ring at position 4 of the thiazole nucleus enhanced the affinity toward the transpeptidase through the formation of additional hydrogen bonds with the residues Arg224 and Asn221. Despite the fact that the in silico results were in line with the results of biofilm analyses, highlighting more favorable binding in the case of *E. faecalis* respect to *S. aureus*, further in vitro tests are warranted in order to confirm this mechanism of action.

## 6 Antibiofilm Compounds

Biofilm is studied as one of the critical bacterial virulence factor responsible for serious chronic infections which proved to be resistant to the majority of antibiotic therapies.^71^

Despite, many investigations focused on the development of novel antivirulence compounds with an antibiofilm mechanism of action,^72,73^ no antibiofilm agents have entered in the clinical practice. This is mainly caused by the lack of in vivo experiments for validating the efficacy of the new agents in preventing biofilm-associated infection.

At present, more than 80% of chronic infectious diseases are biofilm-mediated, therefore new strategies able to combat the formation of biofilm or to eliminate preformed biofilm are urgently needed. The identification of new agents that can inhibit bacterial biofilm but are not affecting microbial growth could lead the development of new antivirulence strategies with reduced selective pressure for the onset of drug resistance.

### 6.1 4-Thiazolidinone Derivatives

**Figure 15:**
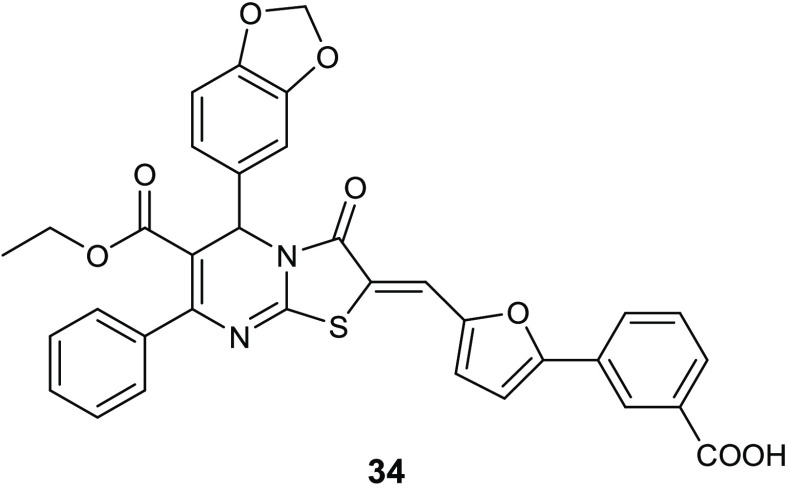
Chemical structures of compound **34**.

Coagulase-negative *S. epidermidis* is currently considered the most common source of infection related to implanted medical devices. The ability of *S. epidermidis* to create biofilms on the facets of medical devices, significantly more resistant to standard antibiotics with respect to the planktonic form, often determine serious chronic infection. Starting from the knowledge on the interesting antibiofilm and antibacterial activity (BIC_50_ = 15.52 μg/mL, MIC = 3.88 μg/mL) of 3-(5-((6-(ethoxycarbonyl)-5-(benzo[1,3]dioxol-5-yl)-3-oxo-7-phenyl-thiazolo[3,2-*a*]pyrimidin-2(5*H*)-ylidene)methyl)furan-2-yl)benzoic acid **34** (Figure 15) against *S. epidermidis*,^74^ Pan and collaborators designed novel thiazolidiones **35** (Table 11) in order to synthesize more effective *S. epidermidis* biofilm inhibitors.^75,76^ The strongest compound, **35e**, resulted in 4-fold more activity in dispersing *S. epidermidis* preformed biofilm (BIC_50_ = 3.08 μg/mL) and in inhibiting bacterial growth of the planktonic form (MIC = 1.54 μg/mL) than the parent compound **34**.

**Table 11:**
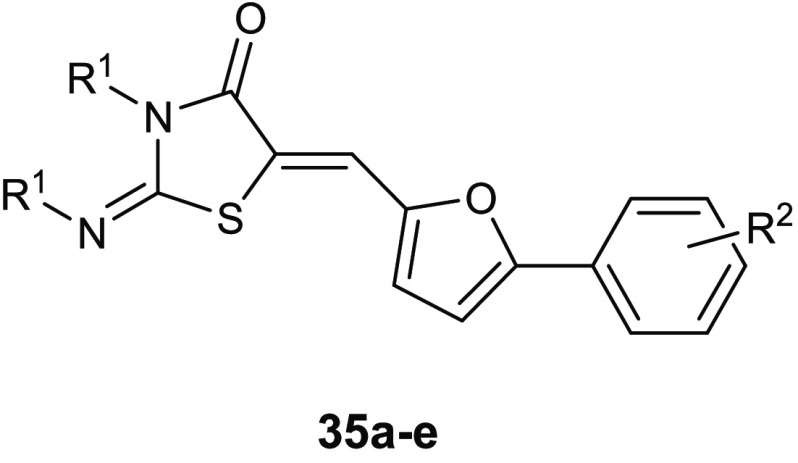
Chemical Structures, Antibacterial Activities (MIC_50_, μg/mL), and Antibiofilm Activities (BIC_50_, μg/mL) of Derivatives **35a**–**e**

compd	R^1^	R^2^	MIC_50_*S. epidermidis*	BIC_50_*S. epidermidis*
**35a**	4-OCH_3_-Ph-CH_2_–	3-COOH	27.73	55.46
**35b**	4-OCH_3_-Ph–	3-COOH	3.29	3.29
**35c**	4-CH_3_-Ph-CH_2_–	3-COOH	3.27	6.54
**35d**	2-CH_3_-Ph-CH_2_–	3-COOH	6.54	13.08
**35e**	2-CH_3_-Ph–	4-COOH	1.54	3.08

The marine alkaloid oroidin showed a BIC_50_ against *P. aeruginosa* biofilms of 190 μM. To create more potent antibiofilm compounds, a series of 4-thiazolidinone derivatives **36** (Table 12) of the marine bromopyrrole was developed and evaluated for both antibacterial and antibiofilm activity against *S. epidermidis* ATCC 12228, *S. aureus* ATCC29213, and *E. faecalis* ATCC 29212. All of these compounds displayed encouraging effects against the tested strains.^77^

**Table 12:**
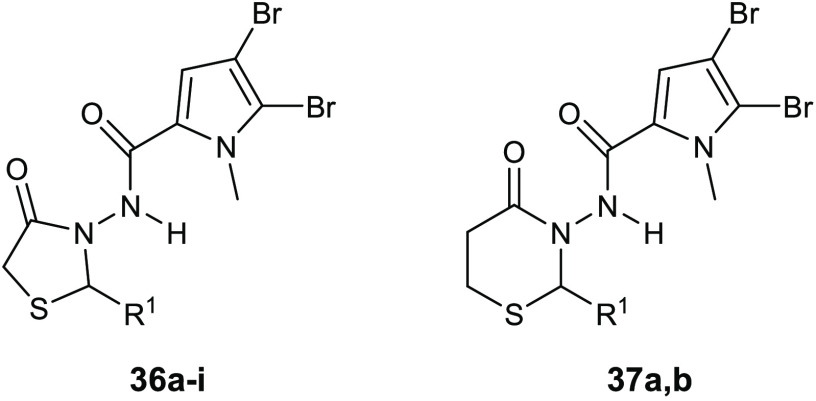
Chemical Structures and Antibacterial Activities (BIC_50_, μg/mL) of Derivatives **36a**–**i** and **37a**,**b**

compd	R^1^	BIC_50_*S. aureus*
**36a**	Ph	3.125
**36b**	4-OCH_3_-Ph	0.78
**36c**	4-NO_2_-Ph–	0.78
**36d**	2-OH,4-OCH_3_-Ph–	1.56
**36e**	3-OH,4-OCH_3_-Ph–	3.125
**36f**	2,5-OH-Ph–	1.56
**36g**	4-F-Ph–	1.56
**36h**	4-Cl-Ph–	1.56
**36i**	cinnamyl	6.125
**37a**	4-OCH_3_-Ph	6.25
**37b**	4-NO_2_-Ph–	6.25

Importantly, compounds **36b** and **36c** were 3-fold more potent than the reference drug vancomycin, demonstrating antibiofilm effects against *S. aureus* ATCC29213 at a concentration of 0.78 μg/mL.

Biological data revealed the importance of the 1,3-thiazolidin-4-one group for the antibiofilm activity, in fact, its replacement with a six-membered ring such as the 1,3-thiazinan-4-ones led to a significant decrease in the activity, as it was demonstrated by the BIC values of compounds **37a**,**b**; the corresponding six-membered analogues of the most active compounds **36b**,**c**.

The condensation of the 4-thiazolidinone ring with a pyrimidine nucleus led to compounds **38a**–**d** (Table 13) that showed antibacterial activity against staphylococcal strains, with MIC values in the range of 0.95–3.82 μg/mL. In addition, all of these compounds exhibited promising antibiofilm activity at 30 μg/mL. The assay of inhibitory activity on YycG histidine kinase suggested that the antibacterial activity of the most promising compound **38a** is based on inhibiting this enzyme (IC_50_ of 7.73 μg/mL), which is an indispensable enzyme in the signal transduction pathway for the cell-wall metabolism.^78^

**Table 13:**
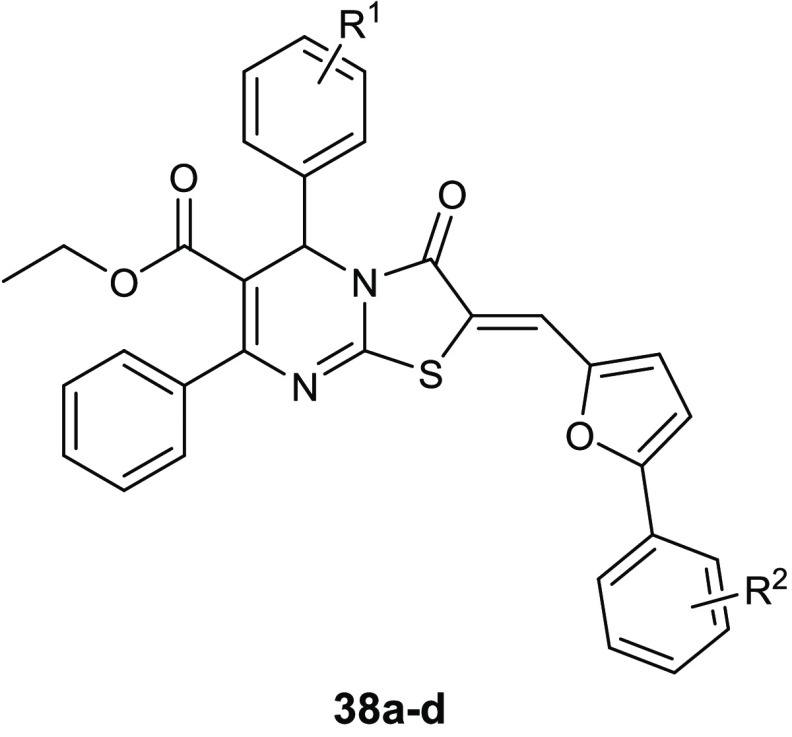
Chemical Structures and Antibacterial Activities (MIC, μg/mL) of Derivatives **38a**–**d**

compd	R^1^	R^2^	*S. epidermidis* ATCC35984	*S.epidermidis* ATCC12282	*S.aureus* ATCC25923
**38a**	4-Cl	3-COOH	0.95	1.91	3.82
**38b**	4-F	3-COOH	3.72	1.86	3.72
**38c**	4-Cl	4-COOH	3.82	1.91	3.82
**38d**	4-F	4-COOH	3.72	3.72	3.72

### 6.2 Thiazoles

**Figure 16:**
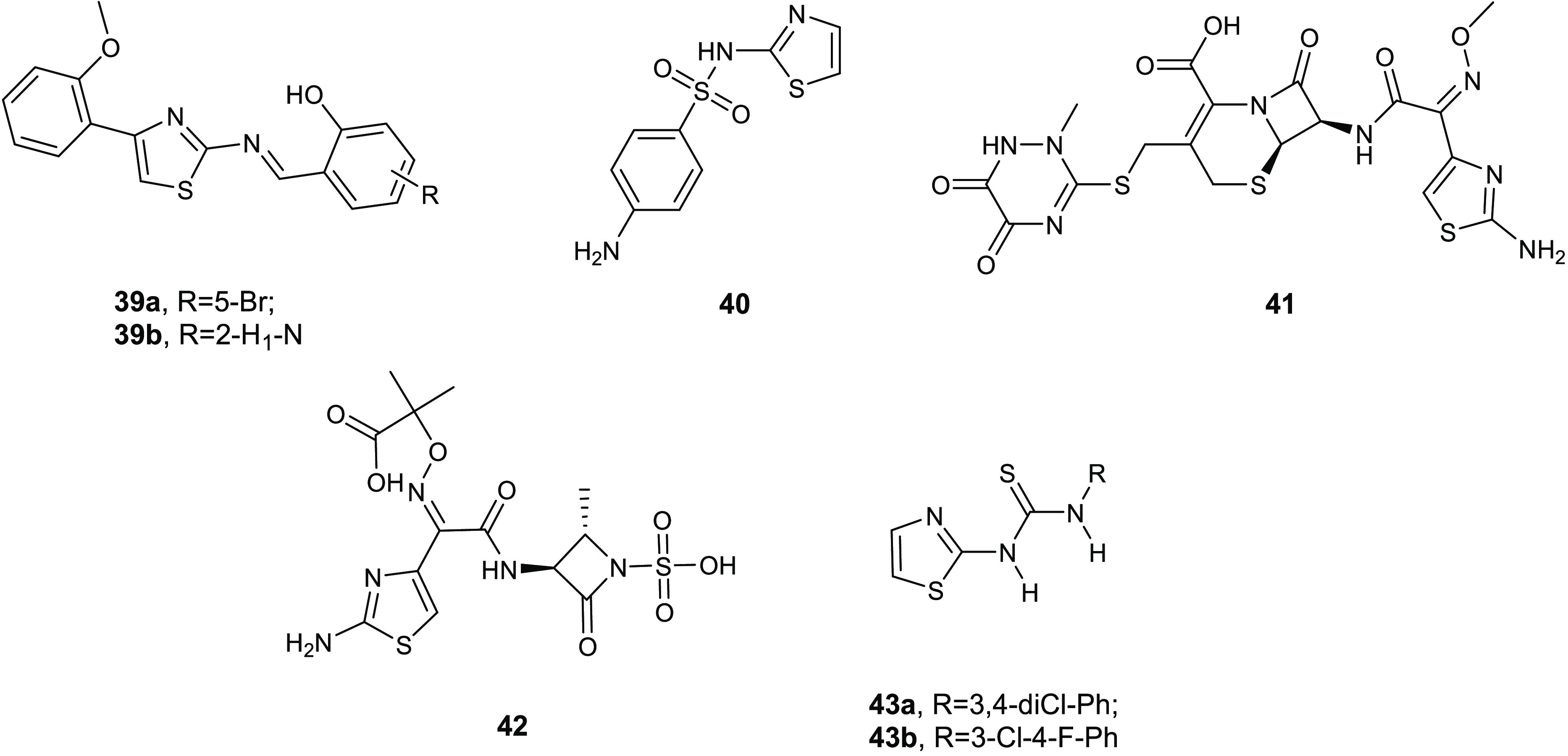
Chemical structures of compounds **39a**,**b**, sulfathiazole (**40**), ceftriaxone (**41**), aztreonam (**42**), and compounds **43a**,**b**.

Because the antibacterial activity of thiazoles and different types of Schiff bases is widely documented,^79,80^ a new series of 4-(*o*-methoxyphenyl)-2-aminothiazoles **39** (Figure 16) was prepared by a microwave-assisted synthesis and evaluated for its antibacterial and antibiofilm effect against *P. aeruginosa*, *Bacillus subtilis*, and *E. coli*.^81^ The best antibacterial activity was observed for compounds **39a** and **39b** against the planktonic form of *B. subtilis*, with MIC values ranging from 25 to 50 μg/mL.

These derivatives were also studied with scanning electron microscope (SEM) and confocal laser scanning microscope (CLSM) analysis in order to evaluate their biofilm inhibition in *P. aeruginosa*, and a significant reduction in the formation of biofilm was found at subinhibitory concentrations. Obtained results suggested a quorum sensing (QS) mediated inhibition of biofilm formation, it being regulated by an intercell communication system aided by released chemical signals.

The QS system is recognized as an appealing target for the development of efficacious antivirulence agents. Because QS is not directly implicated in vital and growth processes of bacteria, its modulation imposes only a minor pressure for the development of mechanisms underlying antibiotic resistance. A therapeutic strategy that fights QS signaling, rather than the bacteria life cycle, may find application in different fields including medicine, agriculture, and food technology.

2-Amino-1,3-thiazole scaffold is known as a qualified pharmacophore for the development of antibacterial agents and other biologically active molecules.^82^ Numerous molecules bearing the 2-aminothiazole structural motif, including sulfathiazole (**40**), ceftriaxone (**41**), and aztreonam (**42**), have reached the clinical or preclinical phase. On the basis of the interesting antibacterial property described for this nucleus, Stefanska and co-workers synthesized a library of novel thiourea derivatives of type **40** (Figure 16), which were examined in vitro against several microorganisms, including relevant Gram-positive and Gram-negative pathogens.^83^

Compounds **43a** and **43b** showed the most promising activity against the staphylococcal planktonic forms eliciting MIC values toward MRSA strains and *S. epidermidis* in the range of 4–16 μg/mL.

The antimicrobial activity is affected by the type and the position of the substituent on phenyl ring. In particular, the 3-chloro-4-fluorophenyl substituted **43b** showed the strongest activity against both standard and hospital Gram-positive pathogen strains.

These thiourea conjugates **43** were further tested, at concentrations 1–16 μg/mL, to evaluate their ability to inhibit the formation of the formation of biofilm in eight methicillin-resistant (MRSE) and two standard (ATCC 12228, ATCC 35984) strains of *S. epidermidis*. In addition to the antimicrobial activity against the free-swimming forms, compounds **43a** and **43b** were the most effective antibiofilm agents, with IC_50_s ranging from 0.35 to 7.32 μg/mL.

Marine natural products (MNP) or marine-derived molecules, such as marine sponge-derived compounds, have been under the spotlight because of their exclusive biodiversity and different structural features as compared to terrestrial products.^84^ Eight of these compounds are used in a number of therapeutic areas, while other compounds are under development in different phases of the clinical pipeline. Only a minority are original MNPs, while most of these compounds are derivatives synthesized through molecular lead optimization.^85^

Bis-indolyl alkaloids, which have two indole units bound to a spacer through their position 3, represent a class of deep-sea sponge metabolites with powerful biological effects, mainly antitumor^86−88^ and antimicrobial properties.^89−94^

Recent studies showed the antibiofilm activity of the new series of bis-indolyl alkaloid nortopsentin analogues **44** in which the imidazole core of the natural product is substituted by the thiazole ring.^95^ Compounds **44a**–**l** (Table 14) did not affect the growth of the planktonic form of the Gram-positive *S. aureus* ATCC 25923, *S. aureus* ATCC 6538, and the Gram-negative *P. aeuruginosa* ATCC 15442 bacteria, showing MICs above 100 μg/mL.

**Table 14:**
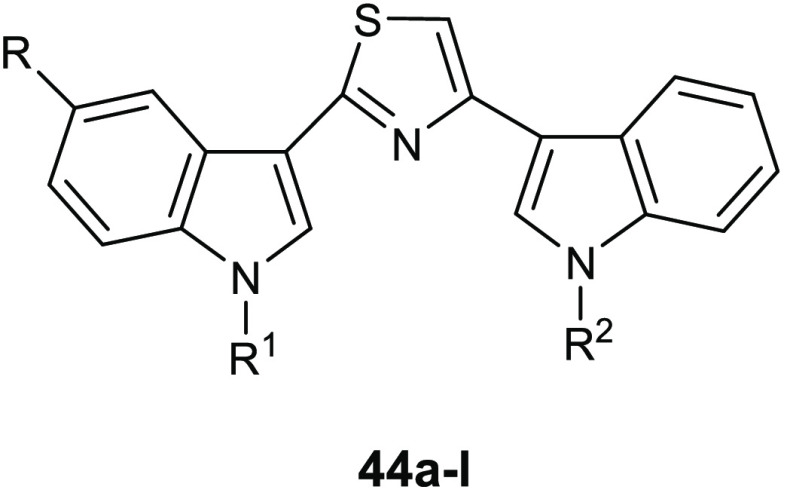
Chemical Structures and Inhibition of Biofilm Formation (IC_50_, μg/mL) of Thiazole Derivatives **44a**–**l**

compd	R	R^1^	R^2^	*S. aureus* ATCC 25923 (μg/mL)	*S. aureus* ATCC 6538 (μg/mL)	*P. aeuriginosa* ATCC 15442 (μg/mL)
**44a**	H	CH_2_CH_2_NHBoc	H	3.9 ± 0.2	5.2 ± 0.3	ns
**44b**	H	CH_2_CH_2_NH_2_	H	4.7 ± 0.3	9.7 ± 0.9	22.7 ± 2.1
**44c**	Br	CH_2_CH_2_NH_2_	H	4.4 ± 0.1	3.3 ± 0.08	7.8 ± 0.09
**44d**	F	CH_2_CH_2_NH_2_	H	1.5 ± 0.1	6.3 ± 0.4	4.5 ± 0.4
**44e**	F	CH_2_CH_2_NH_2_	CH_3_	0.5 ± 0.02	5.2 ± 0.08	3.9 ± 0.07
**44f**	OCH_3_	CH_2_CH_2_OCH_3_	CH_3_	1.2 ± 0.03	11.5 ± 0.7	ns
**44g**	Br	CH_2_CH_2_OCH_3_	H	0.79 ± 0.009	9.4 ± 0.3	4.4 ± 0.08
**44h**	Br	CH_2_CH_2_OCH_3_	CH_3_	0.95 ± 0.01	11.2 ± 1.1	19.1 ± 0.1
**44i**	Br	CH_2_CH_2_OCH_3_	CH_2_CH_2_OCH_3_	2.9 ± 0.02	18.8 ± 1.5	ns
**44j**	Br	CH_3_	CH_2_CH_2_OCH_3_	2.5 ± 0.02	ns	ns
**44k**	F	CH_2_CH_2_OCH_3_	CH_3_	0.2 ± 0.006	21.0 ± 1.7	ns
**44l**	H	Boc	CH_2_CH_2_OCH_3_	1.8 ± 0.1	6.9 ± 0.1	ns

Almost all derivatives were strong inhibitors of the formation of staphylococcal biofilm (Table 14). Thiazole derivatives **44e**, **44g**, **44h**, and **44k** were the most active against *S. aureus* ATCC 25923, showing IC_50_s of 0.5, 0.79, 0.95, and 0.2 μg/mL, respectively.

The lack of activity of the compounds **44** against the planktonic form was desirable in order to obtain antivirulence agents, which cause only a low selective pressure for the development of antibiotic-resistant strains. All of the new compounds were also evaluated at the concentration of 100 μg/mL to examine their capability to disperse preformed biofilm. However, none of these derivatives was able to disrupt biofilm architecture. Compounds **44** interfered with the first step of the formation of biofilm without effecting microbial growth nor preformed-biofilm, showing a significant selectivity against the Gram-positive pathogens respect to the Gram-negative. A mechanism of action involving the inhibition of the transpeptidase SrtA was hypothesized.^96,97^ Compounds **44a** and **44l**, which showed the best biofilm formation inhibitory effects as well as the highest selectivity against Gram-positive pathogens, were chosen to validate this hypothesis. Unfortunately, only compound **44a** was able to moderately inhibit SrtA at the concentration of 100 μM, with a percentage of inhibition around 48%. Therefore, the antiadhesion activity observed for these derivatives was not correlated to the inhibition of the transpeptidase.

### 6.3 Benzothiazoles

The synthesis of hybrid molecules bearing two or more biologically active scaffolds in the same structure, is currently considered a promising approach in order to obtain new therapeutic strategies to treat antibiotic-resistance. The main advantage of this approach consists in the simultaneous presence of two pharmacophores, which can lead to a synergism of the biological activities, thus obtaining molecules able to act toward more than one target.^98,99^

Gondru and co-workers adopted the hybridization approach for the design of the new molecules **45a**–**g** (Table 15), which bear simultaneously in one molecular framework the four pharmacophores pyrazole, thiazole, coumarin, and benzothiazole.^100^

**Table 15:**
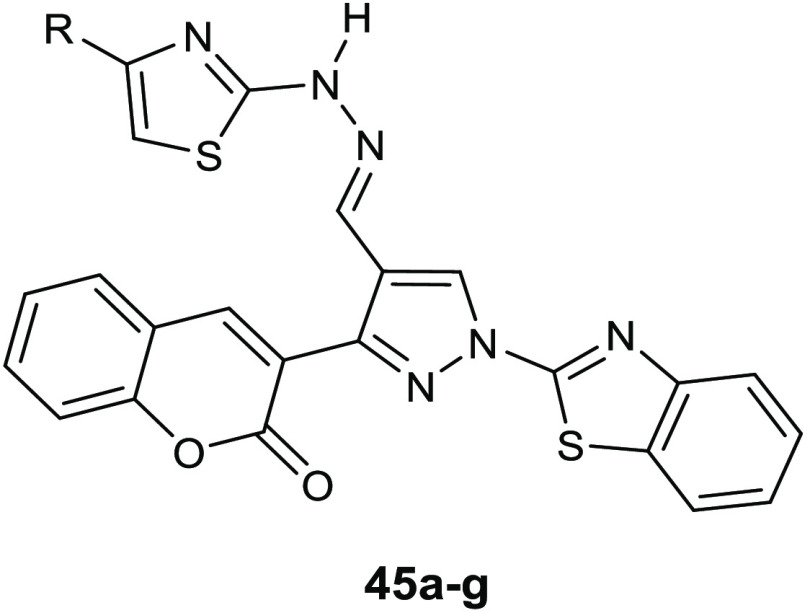

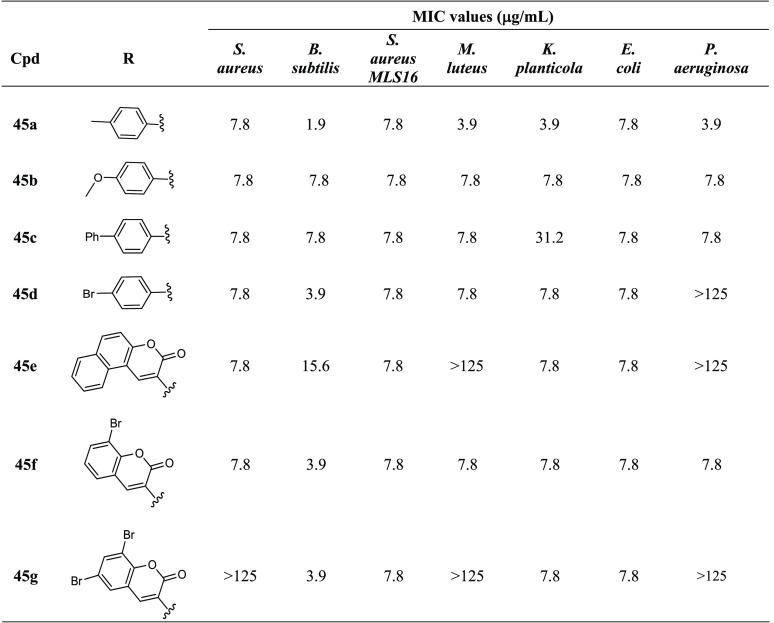
Chemical Structures and Antibacterial Activity of Compounds **45a**–**g**

Compounds **45a**–**g** were evaluated for their antibacterial activity in vitro against Gram-positive (*S. aureus* MTCC 96, *B. subtilis* MTCC 121, *S. aureus* MLS16 MTCC 2940, *Micrococcus luteus* MTCC 2470) and Gram-negative pathogens (*Klebsiella planticola* MTCC 530, *E. coli* MTCC 739, *P. aeruginosa* MTCC 2453), and almost all of these derivatives showed antimicrobial activity at concentrations in the low micromolar range. The strongest derivative **45a** displayed MIC values against all the tested strains in a range of 1.9–7.8 μg/mL. Biofilm inhibition assay revealed promising antibiofilm activity of compound **45e** against *S. aureus* MTCC 96, with a BIC_50_ of 7.8 μg/mL. Additionally, derivative **45f** elicited biofilm inhibitory activity against *S. aureus* MTCC 96, *S. aureus* MLS16 MTCC 2940, *K. planticola* MTCC 530, and *E. coli* MTCC 739, with BIC_50_ values in the range of 8.3–32.6 μg/mL.

The toxicity profile of these compounds was investigated against RAW 264.7 macrophages at concentrations of 2.5× and 10× MIC and, unfortunately, derivatives **45a**–**g** were toxic at the lowest tested concentration. It was hypothesized that the high toxicity of this series was related to the presence of the benzothiazole ring, which undergoes ring-opening generating hydroxylamines with mutagenic and carcinogenic properties. However, this hypothesis was not evaluated in further experimental studies.

GroEL is a homo-oligomeric complex composed of 14 58 kDa subunits arranged in two seven-membered rings stacked back to back, which plays an important role in refolding polypeptides through a process very different from the other molecular chaperones. The folding reaction mediated by GroEL is an ATP-dependent reaction, and it requires a co-chaperone, named GroES.^101^ Because the GroEL/ES system has a key function for bacterial viability, the identification of small molecule able to block such function should be an innovative antibacterial strategy.

With the aim to identify new antimicrobial agents able to perturb protein folding pathways, Kunkle and co-workers performed a high-throughput screen for novel inhibitors of the GroEL/ES folding cycle.^102^ They identified 235 inhibitors of the *E. coli* GroEL/ES chaperonin system and selected a subset of 22 compounds to be further evaluated for their antibacterial activity against the so-called “ESKAPE” pathogens (*Klebsiella pneumonia*, *Acinetobacter baumannii*, *P. aeruginosa*, and *Enterobacter cloacae*).^103^ The results allowed the identification of the benzothiazole **46a** (Table 16) as a hit compound for the synthesis of a novel class of antibacterial agents because it showed comparable bactericidal effects to vancomycin against *S. aureus* without toxic effects on human liver (THLE-3) or kidney (HEK 293) cell lines. Optimization of **46a** has allowed the observation that (i) the presence of a benzothiazole group and the R^2^-hydroxyl are essential for robust inhibition, and (ii) the halogenation at the R^1^ position with a chlorine atom and at R^3^/R^4^ positions with a bromine atom further increases the GroEL/ES inhibitory activity. The strongest compounds of the series (**46a**–**d**) are reported in Table 16. Their effectiveness in the inhibition of GroEL/ES-mediated folding cycle was evaluated by using *E. coli* GroEL/ES as the surrogate chaperonin system for refolding of the enzymes malate dehydrogenase (MDH) and rhodanese (Rho). The results highlighted a strong correlation between the activities toward the two refolding assays, showing in the case of derivatives **46a**–**c** IC_50_ values in the 0.70–4.7 μg/mL range against both systems. Even if studies on the selectivity of these compounds revealed a high affinity also toward the human heat shock protein HSP60/10, the selectivity indices of antibacterial activity and cytotoxicity against human kidney or liver cells were >50-fold.

**Table 16:**
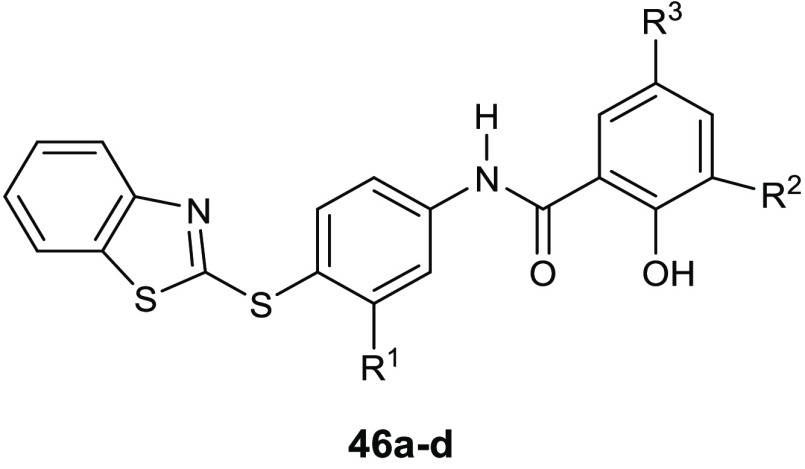
Chemical Structures of Compounds **46a**–**d** and Their EC_50_ Values for Inhibitors Tested in Assays for the Formation of Biofilm and Penetration/Bactericidal Activity

				*S. aureus* proliferation and biofilm assay EC_50_ [μg/mL]		
compd	R^1^	R^2^	R^3^	planktonic growth	preventing biofilm formation	killing bacteria in biofilms
vancomycin				0.97	0.78	>145
**46a**	Cl	Br	Br	0.20	0.41	1.37
**46b**	Cl	Br	H	0.22	0.49	2.75
**46c**	H	Br	Br	0.23	0.29	1.23
**46d**	H	H	H	0.07	0.34	0.76

Compounds **46a**–**d** showed a potent antibacterial activity against the planktonic form of *S. aureus* with EC_50_s in the range from 0.07 to 0.23 μg/mL. Most importantly, the GroEL inhibitor **46a** was particularly effective toward MRSA strain without generating antibiotic resistance when tested following the procedure described by Kim and collaborators to evaluate the ability of compounds to induce drug resistance.^104^

Compounds **46a**–**d** also prevented *S. aureus* biofilm formation eliciting BIC_50_ values of only 0.29–0.49 μg/mL. Being the derivatives equipotent against the planktonic form of the same strain, it is possible that the antibiofilm effect of these compounds is strongly associated with the antibacterial property. Therefore, the authors investigated the ability of this series to kill the bacterial cells inside the biofilm, evaluating their activity on preformed biofilm. In these experiments, compounds **46a**–**d** showed EC_50_ values from 5 to 15-fold higher than the MICs, but they had however higher activity than the reference drug vancomycin.

**Figure 17:**
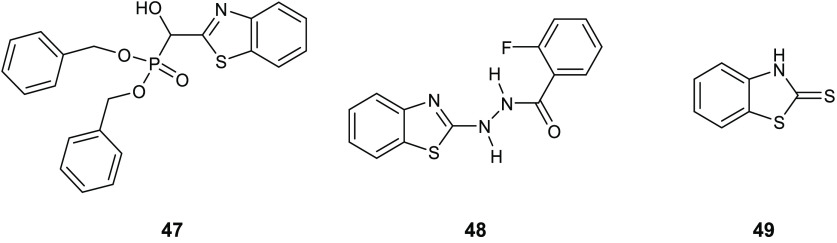
Chemical structures of compounds **47**, **48**, and **49**.

In an attempt to obtain new compounds able to overcome the antibiotic-resistance of *S. aureus*, a dibenzyl (benzo[*d*]thiazol-2-yl(hydroxy)methyl) phosphonate **47** (Figure 17) was prepared and tested for its antibacterial and antibiofilm properties against strains of *S. aureus* resistant to penicillin, ampicillin, and methicillin.^105^ Phosphonates are valuable organophosphorus scaffolds characterized by the presence of a stable C–P bond, which is usually resistant to biochemical and photochemical destruction. The electron withdrawing effects of the thiazole ring may contribute to enhance its stability. The biofilm inhibitory activity of compound **47** was evaluated both as inhibition of biofilm formation and as disruption of preformed biofilm. This phosphonate derivative exhibited an interesting activity against the planktonic form and against the biofilms of *S. aureus* ATCC12600 as well as 12 different drug-resistant strains. It indeed determined the maximum of the activity at the concentration of 160 μg/mL with the total disappearance of bacterial aggregates. Studies on the mechanism of action revealed the lysis of Protein-A (FnBPA), which is an important surface protein belonging to the MSCRAMMs that binds fibronectin and fibrinogen and thus is a key determinant in biofilm formation and colonization.^106^

*P. aeruginosa* is among the most important biofilm-forming strains of Gram-negative pathogens and commonly causes serious chronic infections, especially in the respiratory system of patients suffering from cystic fibrosis. In *P. aeruginosa* biofilms, the matrix is essentially constituted by three different extracellular polymeric substance (EPS) molecules: alginate, Pel, and Psl, which are different for their chemical structure as well as for their biosynthetic mechanisms.^107^

The roles of Pel and Psl in antibiotic resistance, biofilm formation, and immune evasion are well-known. Their overproduction is correlated to a considerable increase in bacterial virulence.^108^ Pel also has important functions in cross-linking eDNA and consequentially in forming the biofilm structure. Deletion of *pelB* results in a severe biofilm decrease, whereas Psl forms fiber-like structures which are fundamental in interactions at cell surface, matrix development, and architecture of the biofilm. The Pel and Psl involvement in antimicrobial resistance has been widely investigated: Pel proved to be crucial for the resistance to aminoglycosides,^109^ while Psl is responsible for the tolerance to polymyxins, aminoglycosides, and fluoroquinolone antibiotics by sequestering them and also by reducing the recognition by the immune system.^110^

With the aim of obtaining novel inhibitors of *P. aeruginosa* biofilm formation targeting Pel and Psl and consequently the EPS secretion, Bernardes and collaborators carried out a high-throughput screen (HTS) for repressors of the gene expression of EPS, *pelB*::*lux*.^111^

Among the *pel* repressors identified, the benzothiazole derivatives **48** and **49** (Figure 17) showed significant antibiofilm activity against PAO1. These compounds were further evaluated for their antivirulence effect against PAO1 in the nematode *Caenorhabditis elegans* slow killing assay. The results of these studies elucidated the capability of these compounds to reduce the virulence of the wild-type PAO1, highlighting the roles of *pel* and *psl* EPS biosynthesis genes as promising tools for the development of antivirulence drugs.

## 7 Thiazoles as Pilicides

Targeting the adhesion of bacteria to host tissue is an emerging antivirulence strategy to counteract the resistance toward antibiotics because this process is necessary for pathogenesis, but it is not fundamental for the microbial growth and viability.^112^

The bacterial cell envelope can be extremely different among bacterial species presenting lipids, proteins, and exopolysaccharides, as well as fimbrial and nonfimbrial structures.

In Gram-negative pathogens, filamentous protein extensions, known as pili, are important virulence factors involved in nonspecific initial adhesion to abiotic surface and tissue colonization.^113^

Among the Gram-negative bacteria, uropathogenic *E. coli* (UPEC) is the primary cause of urinary tract infections (UTIs), which are among the most frequent diseases in community and hospital environments.^114^ In *E. coli*, pilus assembly is mediated by the chaperone/usher pathway (CUP pili), which is granted as a valuable target for the study of antivirulence agents able to prevent *E. coli* adhesion and biofilm formation.

**Figure 18:**
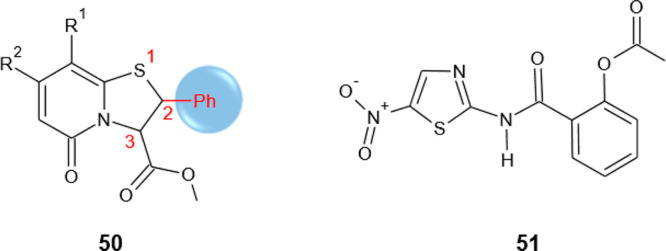
Chemical structures of compounds **50** and nitaxozanide (**51**).

A series of dihydrothiazole derivatives **50** (Figure 18) is described as pilicides able to bind the PapD–PapH chaperone–subunit complex, as revealed by X-ray crystallographic studies.^115^ Many of the C-2 aryl and heteroaryl-substituted derivatives of this class efficiently inhibited *E. coli* UTI89 biofilm formation, showing IC_50_s in the micromolar range, in particular the benzyl substituted displayed the highest potency in the biofilm inhibition evaluation assay with an IC_50_ of 7 μM. The introduction of a phenyl group at C-2 position significantly improved the inhibitory activity of pilus formation due to the establishment of additional hydrophobic interactions with the chaperone shallow pocket formed by the residues Pro30, Leu32, Ile93, and Pro95. The presence of a C-2 phenyl substituent caused a conformational change in the side chain of Leu32 that generated a surface pocket capable of accommodating it.

Recent studies described the pilicides activity of the nitrothiazolyl-salicylamide nitazoxanide (**51**) (NTZ) (Figure 18), which is known for its therapeutic properties toward intestinal diseases including giardiasis and cryptosporidiosis.^116^ NTZ inhibits pili biogenesis by affecting CU pathways. In particular, it was observed that the mode of action is associated with a specific interference with proper maturation of the usher protein in the bacterial outer membrane (OM).

## 8 Compounds with Synergistic Effect in Association with Conventional Antibiotics

Resistance-nodulation division (RND)-type efflux pumps, causing the cellular extrusion of a number of antibiotics, such as β-lactams and β-lactamase inhibitors, fluoroquinolones, tetracyclines, and oxizolidines, play a key role in the MDR phenotype in Gram-negative pathogens.

**Figure 19:**
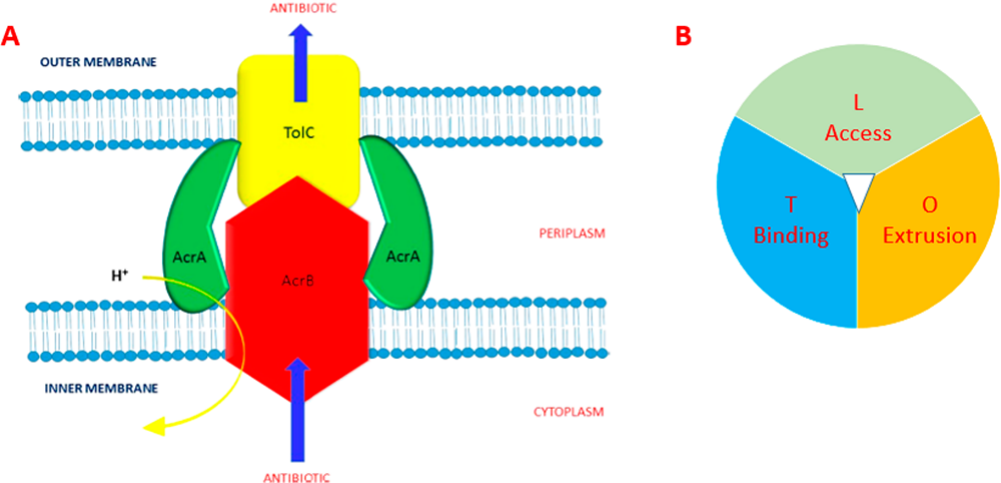
(A) RND efflux pump structure: integral membrane pump protein (AcrB), an outer membrane (OM) channel protein (TolC), and a periplasmic membrane fusion protein (AcrA).^117^ (B) AcrB structure.

Structurally RND pumps are formed by an integral membrane pump protein (AcrB), an OM channel (TolC) and a protein adapter (AcrA) (Figure 19).^117^

Compounds that are able to inhibit efflux pumps are strongly required for restoring or increasing the antimicrobial activity of currently used antibiotics.

The design of new efflux pumps inhibitors (EPI) to overcome the MDR in Gram-negative pathogens have to take into account the structural features of both bacterial OM and RND. Whereas the penetration of the OM is favored for zwitterionic or hydrophilic molecules, contrarily, the binding with RND pumps needs a hydrophobic structure.

Despite many efforts in this field and the development of many EPIs, none of these compounds has reached the clinic to date.

A set of 2-substituted benzothiazoles **52a**–**n** (Table 17) was able to rescue the antibacterial effect of ciprofloxacin (**15**) (CIP) in the AcrAB-TolC overexpressor *E. coli* AG102 mutant.^118^

**Table 17:**
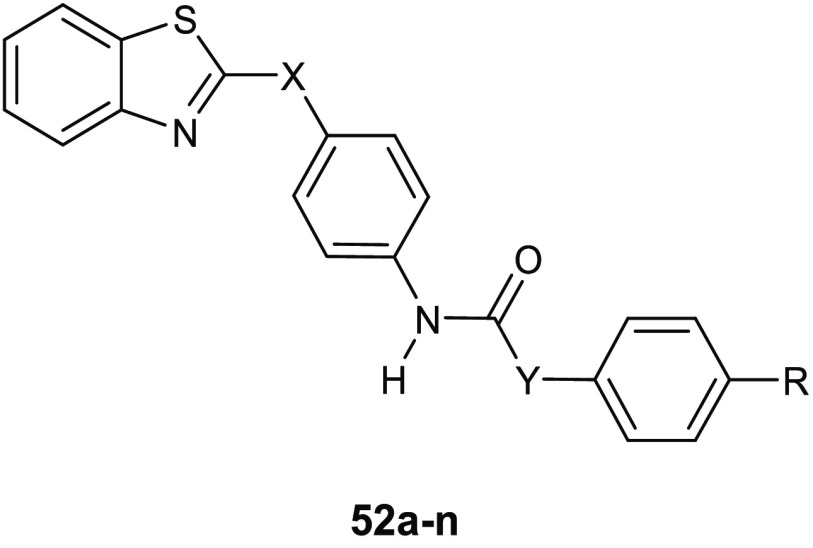
Chemical Structures and Observed MIC Values against the AcrAB-TolC Efflux Pump Overexpressor *E. coli* AG102 Strain of 2-Substituted Benzothiazoles **52a**–**n**

compd	R	X	Y	MIC (μg/mL)^a^	*E. coli* AG102 combination with CIP	MIC (μg/mL)^b^
ciprofloxacin (**15**, CIP)				0.125		
**52a**	H			256	CIP + **24a**	0.03
**52b**	OCH_2_(CH_2_)C_2_H_5_			64	CIP + **24b**	0.5
**52c**	C_2_H_5_			128	CIP + **24c**	0.016
**52d**	OCH_3_		CH_2_	256	CIP + **24d**	0.008
**52e**	F		CH_2_	128	CIP + **24e**	0.03
**52f**	CH_3_		CH_2_	512	CIP + **24f**	0.004
**52g**	H		CH_2_	256	CIP + **24g**	0.125
**52h**	F	CH_2_	CH_2_	256	CIP + **24h**	0.03
**52i**	F	CH_2_		128	CIP + **24i**	0.03
**52j**	Br	CH_2_		128	CIP + **24j**	0.03
**52k**	NO_2_	CH_2_		128	CIP + **24k**	0.06
**52l**	C_2_H_5_	CH_2_		64	CIP + **24l**	0.06
**52m**	H	CH_2_		256	CIP + **24m**	0.016
**52n**	H		C_2_H_4_	512	CIP + **24n**	0.004

a Observed MIC values of compounds tested alone.

b Observed MIC values of CIP tested in combination with each compound.

Derivatives **52f** and **52n** showed a 10-fold reduction in the MIC value of CIP against *E. coli* AG102 strain, while **52d** reduced it by about 8-fold (Table 17). These derivatives did not show intrinsic antibacterial activity but displayed a significant synergistic effect in combination with CIP against *E. coli* AG102. Compounds **52f** and **52n** associated with CIP indeed exhibited the highest antibacterial activity eliciting MIC values of 0.004 μg/mL.

**Figure 20:**
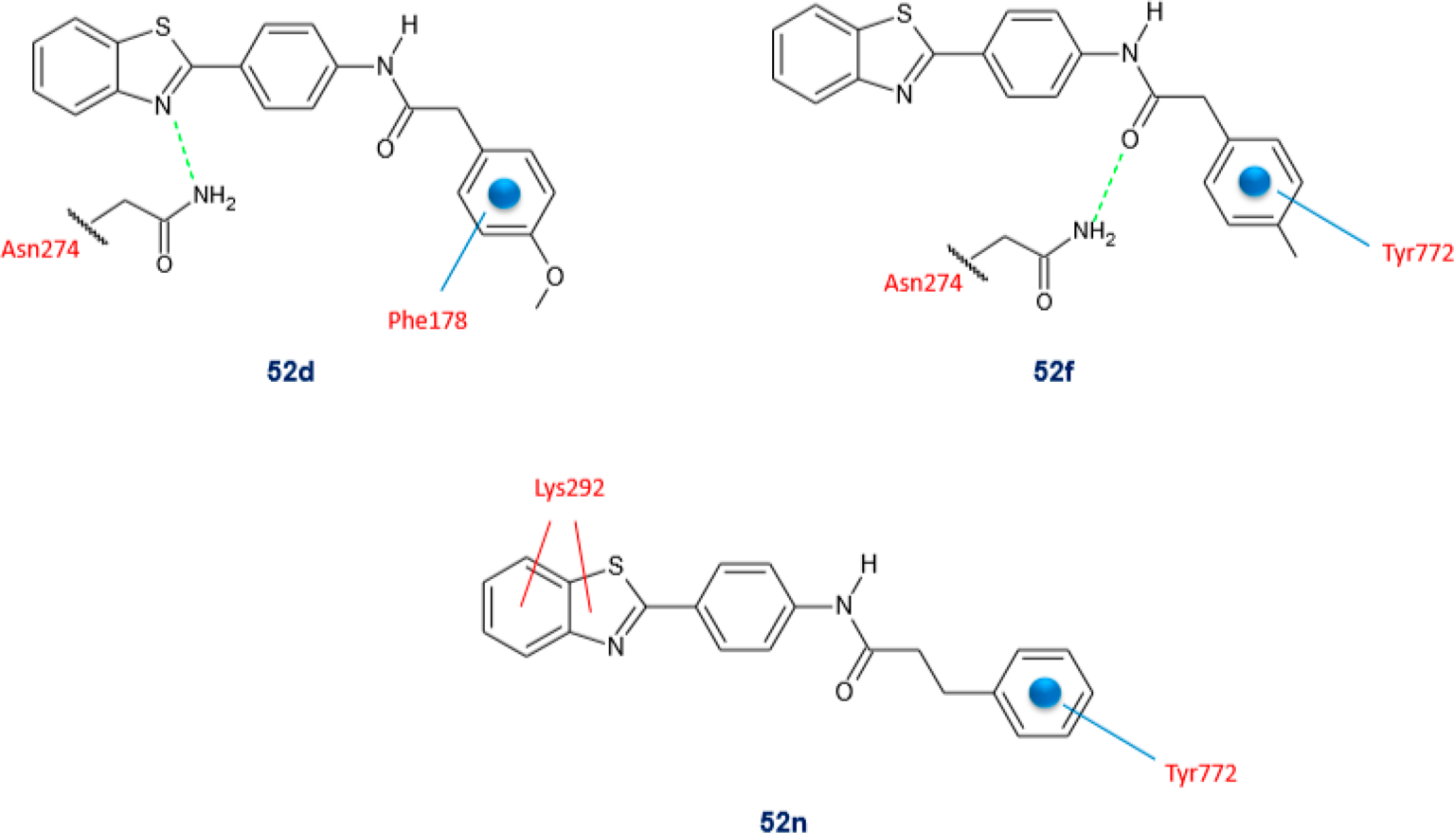
Schematic representation of docking poses of compounds **52d**, **52f**, and **52n** in the AcrB binding monomer crystal structure (PDB 2DRD). Hydrogen bonds in green, a π–π interactions in blue, and π–cation interactions in red.

Results of docking studies performed on **52**, which have low molecular weights (in the range 330–402), in the crystal structure of the binding monomer T of AcrB (PDB 2DRD) were in agreement with the data obtained from the cellular assay. In fact, the major affinity toward the binding site of the monomer AcrB was shown by the derivatives **52d**, **52f**, and **52n**, which displayed stronger binding interactions energies in comparison with CIP. All results suggested a mechanism of inhibition of AcrB by binding to the phenylalanine-rich region in the distal pocket. In particular, derivatives **52d** and **52f** docking poses showed a hydrogen bond with Asn274 and a π–π interaction with Phe178 and Tyr772, respectively. However, compound **52n** showed a π–π interaction with Tyr772 and two π–cation interactions with Lys292 (Figure 20). The three compounds bound to the same region in the deep distal binding pocket of the T monomer, inhibiting or blocking CIP binding site. Studies of the binding energies of **52d**, **52f**, and **52n** showed, for derivatives **52f** and **52n**, stronger binding energies than CIP, while for **52d**, a lower energy was found. Therefore, two different mechanisms were hypothesized: a competitive inhibition of the CIP binding site for compounds **52f** and **52n** and an uncompetitive inhibition for **52d**.

**Figure 21:**
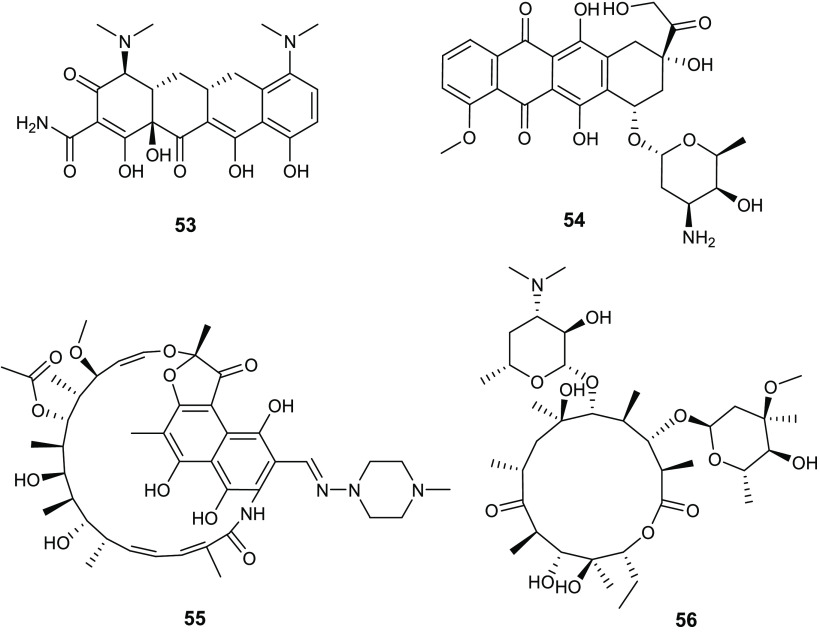
Chemical structures of minocycline (**53**), doxorubicin (**54**), rifampicin (**55**), and erythromycin (**56**).

AcrB is a protein of 1049 amino acids organized in three monomers or protomers characterized by different conformations: (i) loose (L), which is the access protomer, (ii) tight (T), which is the binding protomer, and (iii) open (O), which works as extrusion protomer. Crystallographic studies of AcrB with its substrates minocycline (**53**) and doxorubicin (**54**) highlighted that only one protomer is involved in the binding with the ligand and it depends by the molecular mass of the substrate. Compounds with low molecular mass, including minocycline (**53**) and doxorubicin (**54**), were bound in the T protomer in the phenylalanine-rich region. Conversely, compounds with high molecular mass, such as rifampicin (**55**) and erythromycin (**56**), docked the L monomer. The two different binding pockets are known as distal and proximal, respectively (Figure 21).

Most recently, another class of thiazole derivatives endowed with interesting synergistic effect with conventional antibiotics was discovered within a study aimed to obtain new sulfonamide derivatives as dihydropteroate synthase (DPHS) inhibitors.^119^

The antibacterial activity of sulfonamides is due to the modulation of the folate pathway by inhibiting DPHS, which drives the formation of dihydropteroate (DHPt) from *p*-aminobenzoic acid (PABA) and 6-hydroxymethyl-7,8-dihydropterin-pyrophosphate (DHPPP).

**Figure 22:**
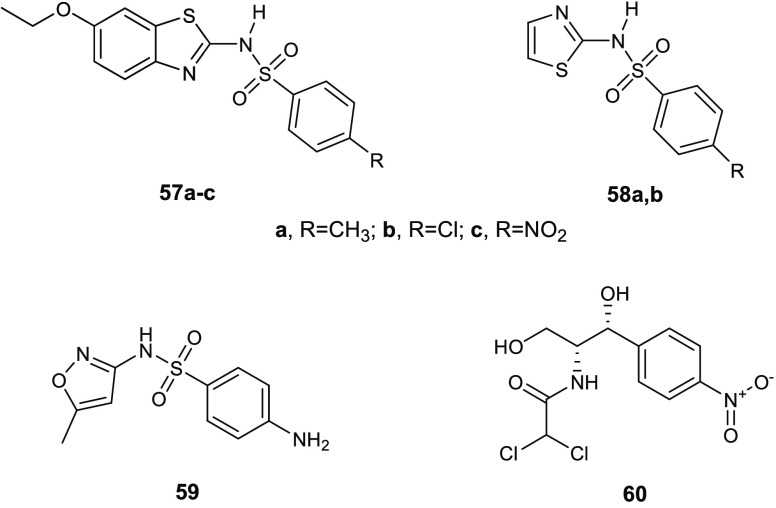
Chemical structures of compounds **57a**–**c**, **58a**,**b**, sulfamethoxazole (**59**), and chloramphenicol (**60**).

Compounds **57a**–**c** and **58a**,**b** (Figure 22) were active against all the tested strains, i.e., *B. cerus*, *S. aureus*, *E. coli*, and *P. aeruginosa* and showed a peculiar selectivity toward the Gram-negative *E. coli*, with MIC values in the range of 3.1–12.5 μg/mL. In particular, when compared with the reference drugs, the thiazole derivative **58a** (MIC, 3.1 μg/mL) was equipotent to sulfamethoxazole (**59**) (Figure 22) and two times more potent than chloramphenicol (**60**) (MIC, 6.2 μg/mL) (Figure 22) against *E. coli* and *P. aeruginosa*. Further studies evaluated the synergistic effect of the new compounds in terms of fractional inhibitory concentration (FIC) through a combination study with sulfamethoxazole (**59**) and chloramphenicol (**60**) against all four bacterial strains. FIC values of 0.24 and 0.25 were observed in all tested strains. The compounds used in association with sulfamethoxazole (**59**) and chloramphenicol (**60**) demonstrated their ability to diminish the MIC of the antibiotic of 8-fold against *E. coli*. Remarkably, the combined MIC value (0.1953 μg/mL) of compound **58b** with chloramphenicol (**60**) was 32-fold lower than the original MIC of 6.2 μg/mL. Such results highlighted the value of these compounds as efficacious adjuvants in an antibacterial combination approach.

## 9 Antibacterial Activity of Thiazole Derivatives with Unknown Mechanism of Action

### 9.1 Thiazoles

**Figure 23:**
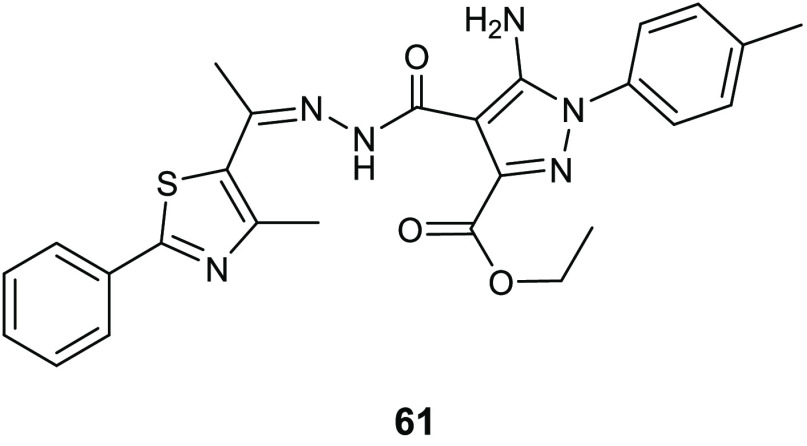
Chemical structure of compound **61**.

Very recently, new thiazole-based compounds, **61**(120) (Figure 23) and **62a**–**g** (Table 18),^121^ were synthesized and tested for their antibacterial activity. The derivative **61** showed MIC values ranging from 78.1 to 396 μg/mL against the Gram-positive *S. aureus* and *Bacillus subtilis* and against the Gram-negative *E. coli* and *Proteus vulgaris* pathogens, resulting in being more efficacious toward *S. aureus*. The presence of *p*-tolyl and ethoxycarbonyl groups at positions 1 and 3, respectively, of the pyrazole ring was advantageous for the antibacterial activity of this class of compounds.

**Table 18:**
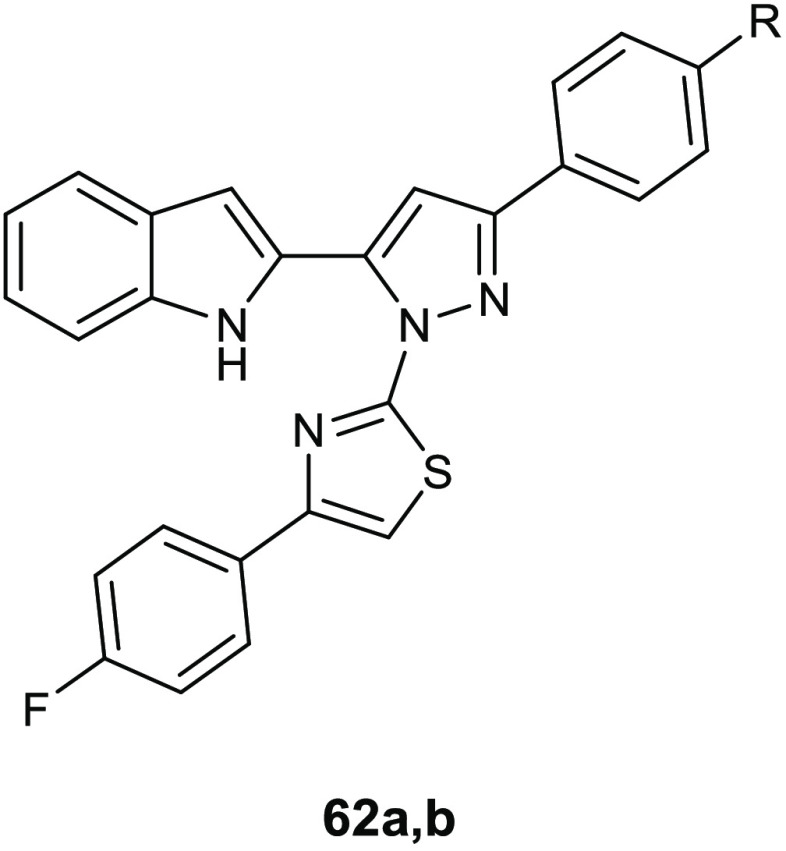
Chemical Structures and Antibacterial Activities of Compounds **62a**,**b**

		MIC values (μg/mL)			
compd	R	*S. aureus*	*B. subtilis*	*P. aeruginosa*	*K. pneumoniae*
**62a**	NO_2_	25	25	6.25	12.5
**62b**	F	50	50	25	25
chloramphenicol (**60**)		6.25	6.25	6.25	12.5

For the thiazole series **62**, the antibacterial activity against *S. aureus*, *B. subtilis*, *P. aeruginosa*, and *K. pneumoniae* was positively influenced by the presence of electron withdrawing groups on the phenyl ring, as it has been confirmed by MIC values in the range of 6.25–50 μg/mL for the compounds **62a**,**b** (bearing a nitro group or fluorine atom, respectively). SAR studies also highlighted the key role of the thiazole ring for the antibacterial activity. The in vitro antimicrobial studies confirmed the compound **62a** exhibit selective activity toward *P. aeruginosa* and *K. pneumonia*, proving to be equipotent to the standard drug chloramphenicol (**60**) (Table 18).

### 9.2 Benzothiazoles

To unravel the SAR of the 2-mercaptobenzothiazole scaffold for the development of antibacterial agents, Franchini et al. studied if the replacement of the hydrogen at the 6-position of the heterocyclic moiety with groups able to form electronic and electrostatic interactions that could be advantageous for the biological properties.

With this purpose, a series of 2-mercaptobenzothiazole derivatives **63** (Table 19) was synthesized and tested for their antibacterial activity against Gram-positive (*S. aureus*, *Bacillus cereus*, *B. subtilis*, *E. faecalis*) and Gram-negative (*E. coli*, *A. baumannii*, *K. pneumoniae*, *P. aeruginosa*) pathogens. Among the new compounds, derivatives **63a**,**b** were the most active; in particular, they showed the highest antibacterial activity against *S. aureus* ATCC 29213 bacteria strain, eliciting MIC values of 3.12 and 12.5 μg/mL. Additionally, compounds **63a**,**b** proved to be active also against the resistant, overexpressing NorA efflux pump, *S. aureus* bacterial strains, demonstrating that they manage to overcome this mechanism of antibiotic resistance.^122^

**Table 19:**
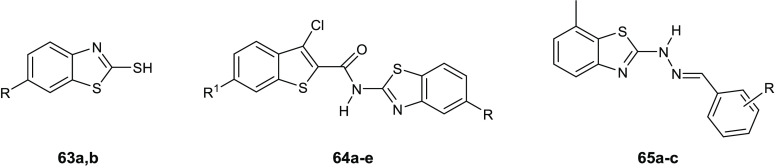
Chemical Structures of Compounds **63a**,**b**, **64a**–**e**, and **65a**–**c**

compd	R	R^1^	MIC (μg/mL)	compd	R	R^1^	MIC (μg/mL)
**63a**	CF_3_		3.12^a^	**64d**	NH_3_^+^Cl^–^	H	8^b^
**63b**	NO_2_		12.5^a^	**64e**	NH_3_^+^Cl^–^	NH_3_^+^Cl^–^	16^b^
**64a**	H	NO_2_	16^b^	**65a**	2,4-diOH-Ph		20^c^
**64b**	H	NH_2_	8^b^	**65b**	2,4-diOCH_3_-Ph		22^c^
**64c**	H	NH_3_^+^Cl^–^	16^b^	**65c**	3,4,5- triOH-Ph		18^c^

a The in vitro minimal inhibitory concentrations (MICs) were determined against *S. aureus* ATCC 29213 bacterial strain.

b The in vitro minimal inhibitory concentrations (MICs) were determined against *E. faecalis* bacteria strain.

c The in vitro minimal inhibitory concentrations (MICs) were determined against methicillin-resistant *S. aureus* (MRSA090) bacterial strain.

Other thiazole derivatives described for their antibacterial activity were the 2-benzothiazolyl benzo[*b*]thieno-2-carboxamides **64a**–**e** (Table 19), which showed interesting activity against *E. faecalis* with MIC values in the range 8–16 μg/mL,^123^ and benzo[*d*]thiazole-hydrazones analogues **65a**–**c** (Table 19),^124^ in which the electron donating groups, such as OH or OCH_3_, play a pivotal role in the antibacterial activity, as it has been confirmed by good MIC values in the range 18–22 μg/mL, obtained for the compounds **65a**–**c** against methicillin-resistant *S. aureus* (MRSA090) bacterial strain.

**Figure 24:**
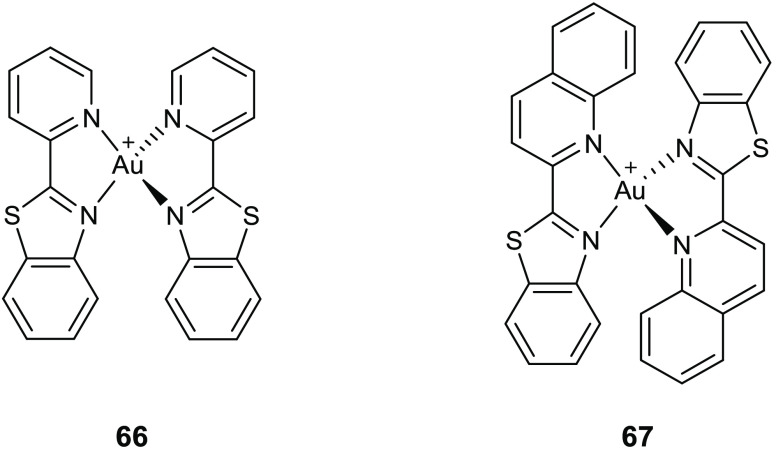
Chemical structures of compounds **66** and **67**.

Also, benzothiazole complexes are reported as potent antibacterial agents. Recently, the antimicrobial properties of two cationic Au(I) complexes derived from aryl-benzothiazoles: [(PPh_3_)Au(pbt)](OTf) **66** and [(PPh_3_)Au(qbt)](OTf) **67** (pbt = 2-(pyridyl)benzothiazole, qbt = (quinolyl)benzothiazole, and OTf = trifluoromethanesulfonate anion) were described (Figure 24).^125^ Complexes **66** and **67** showed strong antibacterial effects against the Gram negative bacteria *A. baumannii* and *P. aeruginosa* in a skin and soft tissue infection (SSTI) model.

Through this model, the gradual penetration of bacteria deeper into the skin was evaluated using a two-layer agar system in which there is a layer of dispersed bacterial cells on the top and a nutrient-rich bottom layer. The gradient determines the slow migration of the bacteria from the top layer to the bottom layer, imitating the infectious process of the skin.

Complexes **66** and **67** were able to migrate and inhibit the infections from *A. baumannii* and *P. aeruginosa* more potently with respect to the neutral starting material PPh_3_AuCl. Results highlighted that the bactericidal effect was mainly due to the interaction between the cationic gold complexes and the bacterial cell membrane. Additionally, they are able to generate the reactive species Ph_3_PAu^+^ inside of the bacterial cell, which binds many biomolecules crucial for bacterial viability. Unfortunately, no information is available on their effects on the onset of antibiotic resistance.

A similar approach was used to obtain a class of imidazo[2,1-*b*]benzothiazolyl triazolium analogues, **68a**–**r** and **69a**–**f** (Table 20), which were tested in vitro for evaluating their antibacterial activity against the Gram-positive pathogens MRSA, *S. aureus*, *B. subtilis*, and *M. luteus* and the Gram-negative *E. coli*, *Shigella dysenteriae*, *P. aeruginosa*, *S. typhi*, and *B. proteus*. Some of them acted as potent antimicrobial agents eliciting MIC values in the low micromolar range.^126^ Of note, the unsubtituted derivatives **68a**–**i** showed higher potency compared to the compounds bearing the ethoxy group at R^1^ position (**68j**–**r**). The series **69** proved to be less effective than **68**, suggesting a detrimental effect of the alkyl chain for the antibacterial activity. It was observed that the biological activity in this series was influenced by the chain length, in fact, the pentyl derivatives **69a** and **69b** showed the highest potency, exhibiting in the case of compound **69b** a MIC value of 8 μg/mL against *S. aureus* and *B. proteus* and of 2 μg/mL against *M. luteus* and *S. dysenteriae*. The presence of longer chains led to a decrease in the growth inhibition. Among the more potent series, **68** compounds substituted with two halogen atoms were more effective than the monosubstituted, in particular, the dichloro derivatives **68g**, **68h**, and **68q** proved to me more potent than chloromycin (**12**), eliciting MIC values against *P. aeruginosa* of 8, 16, and 4 μg/mL, respectively. Interaction studies of calf thymus DNA with the most active compound of the series **68q** elucidated a possible mechanism of action involving the intercalation into the DNA and the consequent block of replication.

**Table 20:**
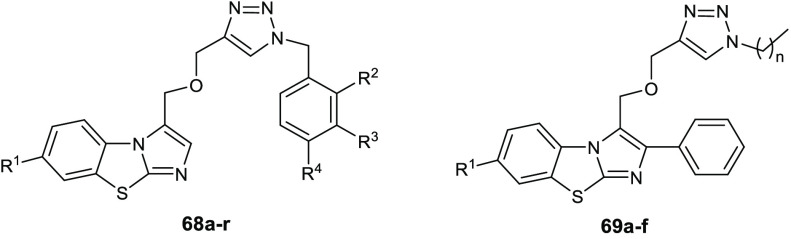
Chemical Structures of Compounds **68a**–**r** and **69a**–**f**

compd	n	R^1^	R^2^	R^3^	R^4^	compd	n	R^1^	R^2^	R^3^	R^4^
**68a**		H	Cl	H	H	**68m**		OEt	F	H	H
**68b**		H	H	Cl	H	**68n**		OEt	H	F	H
**68c**		H	H	H	Cl	**68o**		OEt	H	H	F
**68d**		H	F	H	H	**68p**		OEt	Cl	H	Cl
**68e**		H	H	F	H	**68q**		OEt	H	Cl	Cl
**68f**		H	H	H	F	**68r**		OEt	H	H	NO_2_
**68g**		H	Cl	H	Cl	**69a**	4	H			
**68h**		H	H	Cl	Cl	**69b**	5	H			
**68i**		H	H	H	NO_2_	**69c**	6	H			
**68j**		OEt	Cl	H	H	**69d**	4	OEt			
**68k**		OEt	H	Cl	H	**69e**	5	OEt			
**68l**		OEt	H	H	Cl	**69f**	6	OEt			

### 9.3 4-Thiazolidinone Derivatives

**Figure 25:**
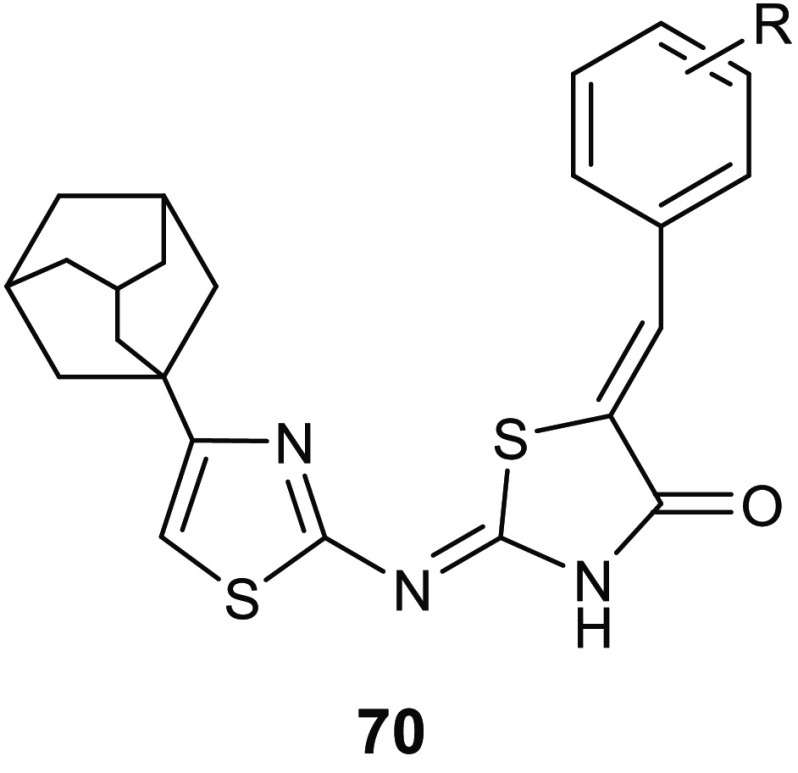
Chemical structures of compounds **70**.

A series of 4-thiazolidinone derivatives of the type **70** (Figure 25), bearing thiazole, thiazolidinone, and adamantane scaffolds, was synthesized and evaluated for the antibacterial activity against a panel of Gram-positive and Gram-negative bacteria including *B. cereus* (clinical isolate), *M. flavus* (ATCC 10240), *L. monocytogenes* (NCTC 7973), *S. aureus* (ATCC 6538), *E. coli* (ATCC 35210), *P. aeruginosa* (ATCC 27853), *S. typhimurium* (ATCC 13311), and *Proteus mirabilis* (human isolate).^127^ All of these compounds elicited a significant antibacterial activity against all the tested strains showing MIC values ranging from 5.01 to 20.8 μg/mL. Determination of MBC, which was almost 2-fold higher than the corresponding MIC, elucidated a bacteriostatic mechanism.

The introduction of arylidene moieties in the thiazolidinone ring improved significantly the antimicrobial activity, and various substitutions on this nucleus were well tolerated. However, despite the excellent antibacterial activity described for derivatives **70** (Figure 25), two critical issues must be taken into account and need further studies: the knowledge of the mechanism of action and the possibility that they were PAINS compounds because of their potential to be Michael acceptor.

Other 4-thiazolidinone derivatives reported for their antibacterial activity were derivatives **71a**–**l** (Table 21), which showed significant properties against the Gram-negative *P. aeruginosa* and *E. coli*, with MIC values ranging from 1.56 to 12.5 μg/mL, and against the Gram-positive *S. aureus* and *B. subtilis*, with MIC values between 1.56 and 6.25 μg/mL.^128^ The highest activity was obtained for derivatives with electron withdrawing group, including a bromine atom or nitro group on the aromatic ring.

**Table 21:**
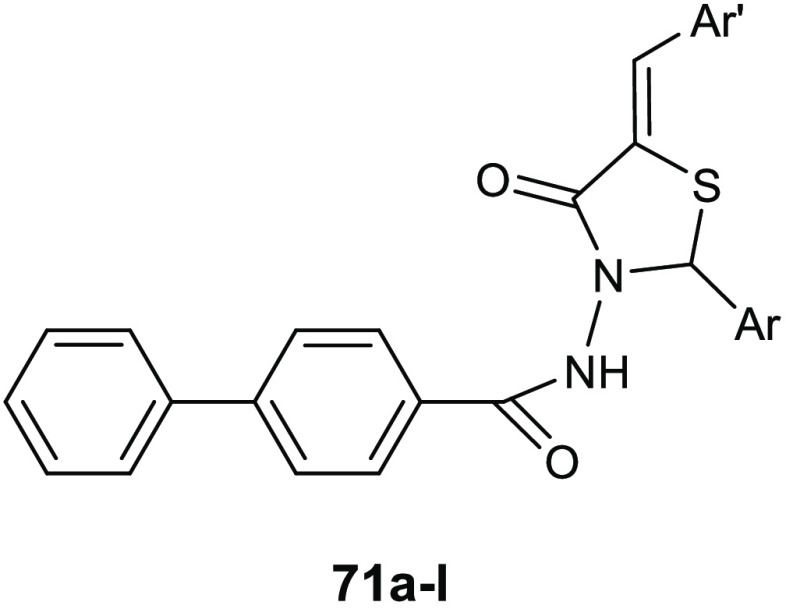
Chemical Structures and Antibacterial Activities of Compounds **71a**–**l**

			MIC values (μg/mL)			
compd	Ar	Ar^1^	*E. coli* MTCC 40	*P. aeruginosa* MTCC 2453	*S. aureus* MTCC 121	*B. subtilis* MTCC 96
**71a**	Ph	Ph	6.25	6.25	6.25	3.12
**71b**	Ph	3-NO_2_-Ph	6.25	3.12	3.12	1.56
**71c**	Ph	4-Cl-Ph	3.12	12.5	3.12	6.25
**71d**	Ph	3-Br-Ph	12.5	6.25	3.12	6.25
**71e**	Ph	3-OCH_3_-Ph	6.25	6.25	6.25	1.56
**71f**	3-Br-Ph	Ph	6.25	3.12	3.12	3.12
**71g**	3-Br-Ph	3-NO_2_-Ph	3.12	1.56	1.56	1.56
**71h**	3-Br-Ph	4-Cl-Ph	12.5	6.25	6.25	6.25
**71i**	3-Br-Ph	3-Br-Ph	6.25	6.25	3.12	3.12
**71j**	3-Br-Ph	4-OCH_3_-Ph	1.56	12.5	3.12	1.56
**71k**	3-F-Ph	Ph	6.25	12.5	6.25	3.12
**71l**	3-F-Ph	3-NO_2_-Ph	12.5	3.12	6.25	6.25

Compounds **72a**–**c** (Table 22), containing the 4-thiazolidinone scaffold, displayed promising antimicrobial activity against the planktonic form as well as the biofilm of *K. planticola*, which is a main responsible cause of nosocomial infections of urinary tract.^129^ These derivatives elicited MIC and MBC values of 3.9 and 15.6 μg/mL, respectively. Additionally, they were able to inhibit *K. planticola* biofilm formation with BIC_50_ ranging from 20.28 to 20.79 μg/mL. The exact target of these molecules is not known, and consequently, the role of the thiazolidinone scaffold for the biological activity is not clear. However, modification on this nucleus influenced the antimicrobial properties. In particular, the presence of electron withdrawing substituents on the thiazolidinone scaffold was advantageous for the antibacterial activity, whereas the substitution of the phenyl ring with different heterocycles caused the loss of this activity. These observations suggest the involvement of the thiazolidinone ring in driving the biological activity.

**Table 22:**
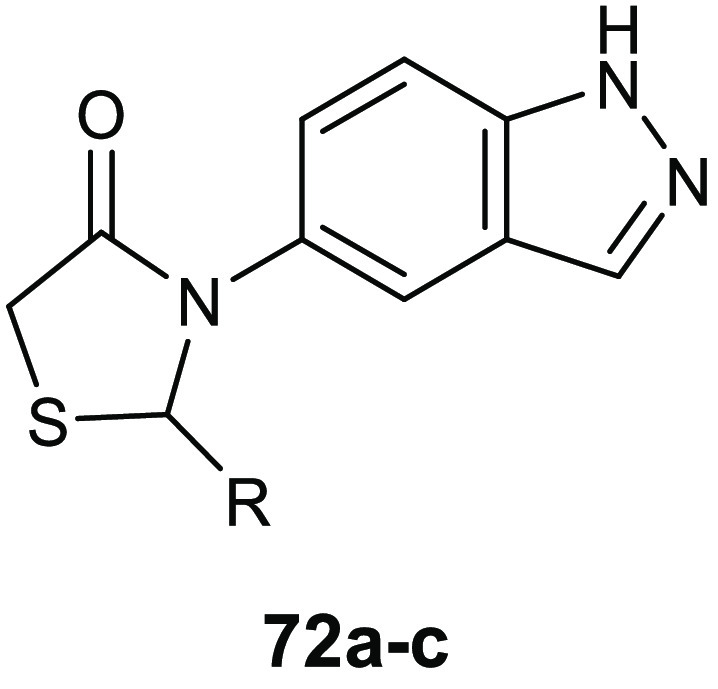
Chemical Structures and Antibacterial Activities of Compounds **72a**–**c**

		*K. planticola* MTCC 530		
compd	R	MIC (μg/mL)	MBC (μg/mL)	BIC (μg/mL)
**72a**	4-CF_3_-Ph	3.9	15.6	20.28
**72b**	3-CF_3_-Ph	3.9	15.6	20.72
**72c**	4-OCF_3_-Ph	3.9	15.6	20.79

Pitta and collaborators combined the three bioactive scaffolds thiazole, adamantane, and 4-thiazolidinone in order to obtain a new series of derivatives **73** (Table 23) with a remarkable antibacterial activity against a wide spectrum of Gram-positive and Gram-negative bacteria.^130^

**Table 23:**
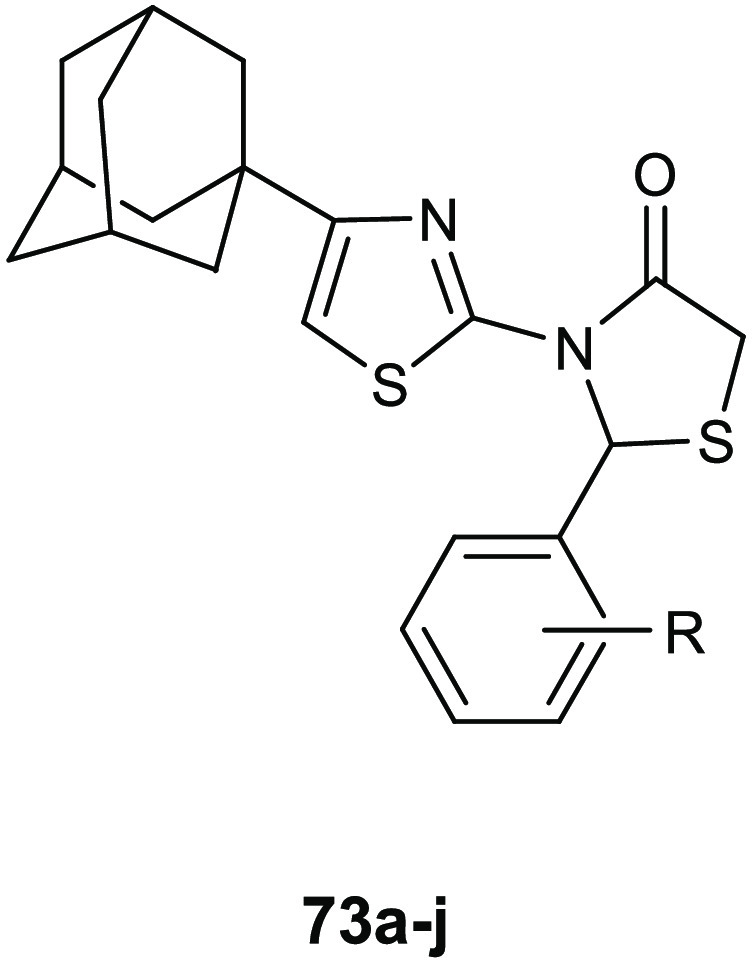
Chemical Structures and Antibacterial Activity of Compounds **73a**–**j**

		MIC values (μg/mL)							
compd	R	*B. cereus*	*M. flavus*	*S. aureus*	*L. monocytogenes*	*E. coli*	*E. coclae*	*P. aeruginosa*	*S. typhimurium*
**73a**	2-Cl	13.2	19.4	26.9	7.0	26.9	13.2	26.9	26.9
**73b**	3-Cl	7.0	26.9	13.2	26.9	26.9	26.9	29.6	13.2
**73c**	4-Cl	7.0	13.2	7.0	26.9	26.9	26.9	7.0	7.0
**73d**	2,3-diCl	14.2	14.2	4.18	29.0	29.0	14.2	7.1	7.1
**73e**	2,6-diCl	200	100	200	200	100	200	200	100
**73f**	3-F	124	12.7	124	6.33	6.33	12.7	3.7	3.7
**73g**	4-Br	47	100	200	200	100	9.5	200	100
**73h**	4-NO_2_	7.2	27	13.5	27	37.5	13.5	13.5	13.5
**73i**	4-OCH_3_	6.9	26.6	13.0	26.6	13.0	13	13.0	13.0
**73j**	2,5-diOCH_3_	100	100	200	200	100	100	200	100

These compounds were more potent than the references drugs ampicillin and streptomycin, showing MIC values ranging from 3.7 to 200 μg/mL against the Gram-positive *Bacillus cereus* (clinical isolate), *Micrococcus flavus* (ATCC 10240), *S. aureus* (ATCC 6538), and *Listeria monocytogenes* (NCTC 7973), and the Gram-negative *E. coli* (ATCC 35210), *Enterobacter cloacae* (human isolate), *P. aeruginosa* (ATCC 27853), and *Salmonella typhimurium* (ATCC 13311).

The highest antibacterial activity was found for compound **73d**, which elicited MIC values in the range of 4.18–29 μg/mL against all the tested strains. Notably, the position of the chlorine atom on the phenyl ring did not influence the biological activity of this class of compounds, whereas the introduction of a second chlorine was advantageous for the activity in the case of 2,3-disubstituted compounds but was detrimental in the case of the 2,6-disubstituted derivative. The presence of a fluorine atom at position 4 in the phenyl ring led to an improvement in the antimicrobial activity against all the bacterial strains except for *B. cereus* and *S. aureus*.

To investigate the potential mechanism of action involving the enzyme MurB, the most active compounds **73d** and **73e** were docked in the *S. aureus* MurB active site (PDB 1HSK).

Compound **73d** (*R*-isomer) formed hydrogen bond contacts with the residues Gly249 and Arg242, and it was also involved in a Cl−π interaction with Phe274 of the active site. The phenyl ring of the 2,3-dichlorophenyl moiety is proposed to be involved in hydrophobic interactions with the amino acids Val239 and Gly273 and in π–π interaction with Phe274. Although in silico studies suggested a good affinity between derivatives **73** and MurB, no in vitro experiments on the enzyme have been carried out to validate this hypothesis.

A new series of 3-(2*H*-1,2,4-triazol-5-yl)-1,3-thiazolidin-4-one derivatives **74a**–**e** (Table 24) was created and tested in vitro for the antibacterial activity and in silico for the MurB affinity.^131^ The highest activity was observed for derivative **74a**, which showed MIC values of 8 μg/mL against *S. aureus* and 16 μg/mL against *E. faecalis*, *B. subtilis*, and *E. coli*. This was attributed to the good lipophilic features of this compound (log *P* value of 3.84), which makes possible the crossing of the lipid coat of bacteria. SAR studies highlighted that the presence of a methoxy group at the R position and fluorine, nitro, methoxy, and methyl at the R1 are advantageous for the antibacterial activity of these compounds.

**Table 24:**
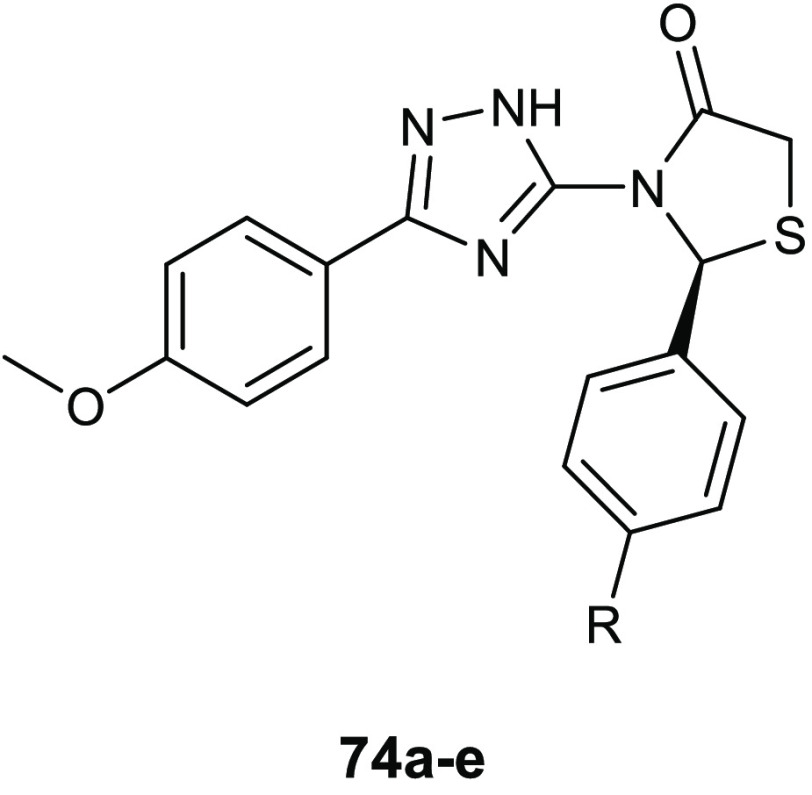
Chemical Structures and Antibacterial Activity of Compounds **74a**–**e**^a^

		MIC values (μg/mL)			
compd	R	*S. aureus*	*E. faecalis*	*B. subtilis*	*E. coli*
**74a**	Cl	8	16	16	16
**74b**	F	128	ND	ND	64
**74c**	NO_2_	16	ND	ND	32
**74d**	OCH_3_	128	ND	ND	6
**74e**	CH_3_	64	ND	ND	32

a ND = not determined.

Studies of molecular modeling with **74a** revealed a good affinity toward the enzyme MurB with a binding energy of −12.18234 kcal/mol, but also in this case, the computational results are not supported by enzymatic assays.

A series of 4-thiazolidinone derivatives **75a**–**e** (Table 25) was described for their potent antimicrobial activity against *S. aureus*, *B. subtilis*, and *E. coli*, showing comparable potency to norfloxacin used as reference drug.^132^ The highest activities were elicited by compounds **75b** and **75d**, against the Gram-negative *E. coli* pathogen, with a MIC value of 3.07 μg/mL, and against *S. aureus*, with a MIC value of 6.51 μg/mL, respectively. Against Gram-positive *B. subtilis* pathogen, the derivative **75a** showed the best MIC value of 6.24 mg/mL. The new compounds showed a bacteriostatic activity because their MBC values were 3-fold higher than the MICs. The introduction of electron withdrawing groups increased the antibacterial activity of this class of compounds against *S. aureus* and *B. subtilis*, while electron donating groups were advantageous for the activity against *E. coli*.

**Table 25:**
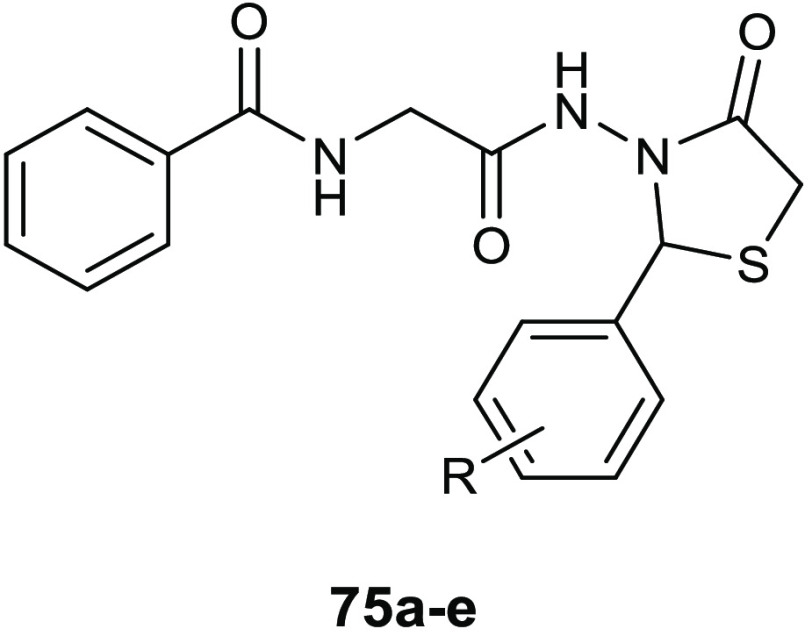
Chemical Structures and Antibacterial Activities of the 4-Thiazolidinones **75a**–**e**

		MIC (μg/mL)		
compd	R	*S. aureus*	*B. subtilis*	*E. coli*
**75a**	2-Cl	12.5	6.24	6.24
**75b**	4-Br	6.51	47.8	12.16
**75c**	3-NO_2_	25.23	12.41	25.23
**75d**	4-N(CH_3_CH_2_)_2_	12.37	12.37	3.07
**75e**	4-CH_3_	12.56	12.56	12.56

**Figure 26:**
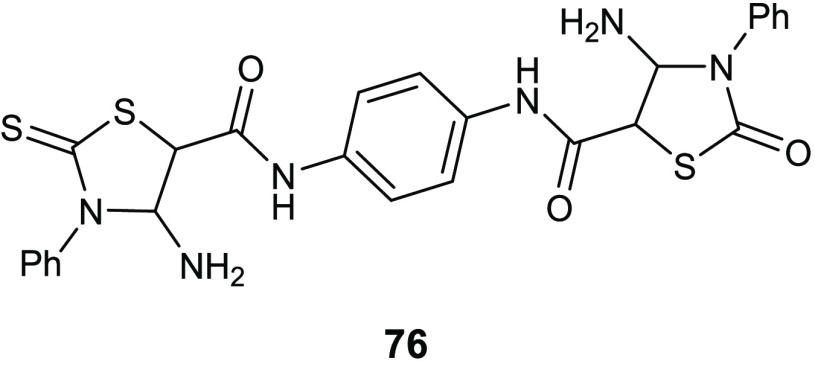
Chemical structure of **76**.

Recently, a sulfurated analogue of thiazolidinone compounds, the 1,3-thiazolidin-2-thione **76** (Figure 26), was found to act as an antibacterial agent equipotent to chloramphenicol (**60**) against *S aureus*, with a MIC value of 3.12 μg/mL, and with a significant activity also against *Bacillus thuringiensis*, with a MIC value of 6.25 μg/mL.^133^ Despite this interesting activity, thiazole derivative **76** did not demonstrate a significant increase in activity with respect to the pyridine analogue previously reported. Conversely, the authors observed a decrease in the activity against the Gram-negative pathogens was. Therefore, in this case, the presence of the thiazole scaffold seems not relevant for the antibacterial activity.

## 10 Conclusions and Future Directions

There is a need of a prompt intervention to face the global emergency of antibiotic resistance.

In addition to the rational use of the conventional antibiotics, which requires coherent strategy in human, animal, plant, and environmental health (One Health strategy), in order to preserve their efficacy in treating infectious diseases, other actions, such as development of new antimicrobial agents, are needed.

In the past decade, many thiazoles and benzothiazoles were described for their interesting antibacterial activity, in many cases also against MDR strains. One of the most significant feature of this class of compounds consists in their ability to interfere with diverse bacterial targets, including DNA gyrase, topoisomerase IV, biofilm formation, cell wall permeability, and tryptophanyl-tRNA synthetase. Consequently, they proved to be active against a broad spectrum of relevant Gram-positive and Gram-negative pathogens, with MIC values in the low micromolar range. Regarding the mechanisms of action, the most investigated is the inhibition of type II topoisomerases. In particular, the benzothiazoles **3a**,**b** potently inhibited in vitro *E. coli* DNA gyrase and topoisomerase IV, with IC_50_ in the range 0.0033–0.046 μg/mL, eliciting MIC values between 0.008 to 0.06 μg/mL against *S. pneumoniae*, *S. epidermidis*, and *S. pyogenes*. In addition, because DNA gyrase is a crucial enzyme for bacterial viability, its inhibition could cause the development of antibiotic resistance strains, as was observed for the fluoroquinolone antibiotic class. Therefore, particularly relevant has been the study performed for evaluating their ability to induce resistance in terms of FoR, which established that these benzothiazole ethyl urea derivatives had a low capability to determine drug resistance, then they could be considered interesting lead compounds worthy of being developed.

Topoisomerases can be considered valuable targets for drug discovery as they are conserved across numerous bacterial species, so a broad spectrum of activity should be feasible. Moreover, in the past decade, new insights have been obtained in the molecular mechanisms of DNA gyrase and topoisomerase IV inhibition, the individuation of the structural features required for the interaction with the binding site and the factors responsible for the resistance development. This knowledge allowed identifying clinical drug candidates such as AZD5099 (**19**), which proved to be efficacious in different in vivo models for the treatment of nosocomial lung and skin infections caused by relevant Gram-positive pathogens.

Importantly, AZD5099 (**19**) showed FoR lower than the detection limit of 9.6 × 10^–10^ and impressive selectivity against the bacterial topoisomerase II compared to the human enzyme.

The modulation of the bacterial cell wall synthesis and permeability still remains a good target for the development of innovative antimicrobials, and many thiazoles are able to inhibit enzymes involved in these processes. However, assays to investigate the FoR values should be carried out because targets responsible for the integrity of the cell wall are needed for the bacterial viability, consequently the evolution of antibiotic resistance is unavoidable. Particularly relevant the data observed for the cephalosporin Cefiderocol, whose effectiveness and safety has been demonstrated for the treatment of cUTI and acute uncomplicated pyelonephritis in a clinical phase II trial.

An important aspect to take into consideration during the design of new antibacterial compounds bearing the thiazole scaffold is the classification of 14 subclasses of 2-aminothiazoles (2-ATs) as PAINS.^134^ PAINS compounds are promiscuous molecules that give multiple positive results, often not reliable, to different biological assays, and for this reason they should be removed from screening libraries and biological assay.^135,136^ There are different reasons for their promiscuous behavior, including their photoreactivity, the presence of impurities generated by bromomethyl ketones often used as chemical precursors in their preparation, and their chemical feature as thiol-reactive species.^137^ On the other side, it must be considered that the biological and therapeutic value of 2-ATs is widely recognized because many marketed drugs, including antibiotics like cefepime, cefetamet, cefoselis, cefotaxime, cefotiam, cefpodoxime, cefpirome ceftazidime, ceftibuten, and ceftriaxone are bearing this fragment in their structure.

Also, for the thiazolidinone derivatives, already known for their interesting pharmacological profile, it is important to consider, in the design of new antibacterial agents, their potential as PAINS. Classes such as 5-ene-4-thiazolidinones, indeed, often showed good activities within a range of different assays and against numerous proteins. Therefore, studies to exclude that the results are not related to the nature of Michael acceptors are needed.

It is important to consider that the possibility of being Michael acceptors is not necessarily associated with the behavior as PAINS compounds and it is often not confirmed experimentally under physiological conditions.^138^ Additionally, advantageous toxicological profile of several classes of 4-thiazolidinones strongly encourage their development and study as new drugs.^139^

The thiazole derivatives, which hit virulence factors, but do not interfere with the microbial growth, could be considered “evolution proof” and for sure worthy to be developed as antivirulence agents. Among them, the inhibitors of biofilm formation, compounds **44** and **46**, that potently inhibited biofilm formation of relevant pathogens without affecting their viability, can be considered deserving for the design of novel antivirulence compounds.

## Abbreviations Used

AMR: antibiotic-resistance
2-ATs: 2-aminothiazoles
AUC: area under the curve
CLSM: confocal laser scanning microscope
CIA: critical important antimicrobials
CIP: ciprofloxacin
CUP: chaperone/usher pathway
DHPPP: 6-hydroxymethyl-7,8-dihydropterin-pyrophosphate
DHPt: dihydropteroate
DPHS: dihydropteroate synthase
EPI: efflux pumps inhibitors
EPS: extracellular polymeric substances
FBLG: fragment-based lead generation approach
FIC: fractional inhibitory concentration
FnBPA: fibronectin binding protein A
FoRs: spontaneous frequencies of resistance
HTS: high-throughput screening
LPS: lipopolysaccharide
MIC: minimum inhibitory concentrations
MurB: uridine diphosphate-UDP-*N*-acetylenolpyruvylglucosamine reductase
NTZ: nitazoxanide
OM: outer membrane
OTf: trifluoromethanesulfonate anion
PABA: *p*-aminobenzoic acid
pbt: 2-(pyridyl)benzothiazole
PAINS: pan assay interference compound
PK: pharmacokinetic
qbt: (quinolyl)benzothiazole
QS: quorum sensing
SEM: scanning electron microscope
SrtA: sortase A
TrpRS: tryptophanyl-tRNA synthetase
UPEC: uropathogenic *Escherichia coli*
UTIs: urinary tract infections
